# Bayesian reversible-jump for epistasis analysis in genomic studies

**DOI:** 10.1186/s12864-016-3342-6

**Published:** 2016-12-09

**Authors:** Marcio Balestre, Claudio Lopes de Souza

**Affiliations:** 1 Department of Statistics- Federal University of Lavras, Lavras, MG CP 3037 Brazil; 2 Departmento de Genética, Escola de Agricultura Luiz de Queiroz, Universidade de São Paulo, (ESALQ-USP) Piracicaba, São Paulo, 13400-970 CP 83 Brazil

**Keywords:** Bayesian analysis, Maize, QTL, Genome-wide studies

## Abstract

**Background:**

The large amount of data used in genomic analysis has allowed geneticists to achieve some understanding of the genetic architecture of complex traits. Although the information gathered by molecular markers has permitted gains in predictive accuracy and gene discovery, epistatic effects have been ignored based on exhaustive searches requesting estimates of its effects on the whole genome. In this work, we propose the reversible-jump technique to estimate epistasis in the genome without drastically altering the model dimension. To this end, we used a real maize dataset based on 256 F_2:3_ progenies plus a simulation data set based on 300 F_2_ individuals. In the simulation scenario, six QTL presenting main effects (additive and dominance) were combined with seven other epistatic effects totaling 13 QTL controlling the trait.

**Results:**

Our model explored 18,624 candidate epistases, but even in this vast space, only one spurious interaction was found. The three epistases selected by our model, named here as 18x26, 56x68 and 59x93, were very close to simulated ones (19x25, 54x72, 59x91 and 59x94). In the real dataset, we estimate 33,024 epistatic effects, and several minor epistatic combinations were found to explain a significant proportion of the genetic variance. The broad participation of epistasis in the real dataset may indicate the presence of pervasive epistasis acting on maize grain yield.

**Conclusions:**

The power of selecting true epistasis in thousands of possible combinations suggests the attractiveness of our model to handle genomic data

**Electronic supplementary material:**

The online version of this article (doi:10.1186/s12864-016-3342-6) contains supplementary material, which is available to authorized users.

## Background

The contribution of epistasis on the genetic architecture of complex traits has been the focus of recent research in genetics, animals and plant breeding [26]. It has been widely discussed by Carlborg and Haley [5] and Mackay [20, 21] that there is evidence of strong participation of epistasis on the inheritance of quantitative traits. However, as emphasized by these authors, the search for statistical models that depict this genetic phenomenon in an efficient way is very challenging, whether by the supposition about the epistasis distribution in the genome or by its definition, which is exceptionally controversial [6].

Regarding the definition of epistasis, this term was initially described by Bateson [2] to explain the observed deviation from the expected Mendelian segregation, where an allele from a particular locus might interact with other alleles at different loci. In other words, instead of occurring only in intra-locus interactions, Bateson suggested that alleles from different loci may interact with each other to under- or overexpress particular genes. Although the above definition is very simple, it was critical in genetics research, and starting from this claim, several others definitions emerged [29].

Currently, there are different interpretations of epistasis, and these divergences have caused some confusion [6]. For example, geneticists have used the term “epistasis” to describe three different events: i) the functional relation among two or more genes, ii) genetic ordering in regulatory pathways and iii) deviation from the additivity in the effect of alleles at different loci in relation to their contribution to a quantitative phenotype. In a similar way, Phillips [29] classified three types of epistasis: i) functional or molecular epistasis, where proteins or other gene products from different genes interact with each other in metabolic pathways, ii) compositional epistasis, which is related to the classical view of epistasis, where one allele from a specific locus can interfere with the expression of other loci, namely, it is individual-specific, and iii) statistical epistasis, which Sir Ronald Fisher adopted as an average deviation from allelic combination expected by Mendelian segregation for different loci in a population. This last one is the most studied in quantitative genetics due to the high genetic complexity involving quantitative traits, and therefore, the estimates of compositional epistasis become unfeasible in traits controlled by several loci [29].

Regarding the distribution of epistasis in the genome, it has been suggested that there is a “universal epistasis”; in other words, gene interactions could be more pervasive than supposed by quantitative geneticists, playing an important role in the genetic control of the traits [3]. For example, these authors observed that more than half of the QTL effects found in yeast mapping were modified by other QTL, presenting marginal or non-significant effects. This result indicates that mapping studies may underestimate the real role of epistasis in complex traits [17].

Mackay [20, 21] presented a similar discussion about the importance of epistasis, highlighting the hypothesis of “universal epistasis” as a nuisance for specific epistasis detection in statistical models. In both works, Mackay suggests the influence of “pervasive” epistasis on so-called “missing heritability”; namely, considering only additive effects and their interactions, the observed broad-sense heritability may be much higher than expected. In this case, artificial selection could be challenging, and the introduction of new alleles in a new background may lead to results that are contrary to those expected [21]. Moreover, the genome selection based on the prediction of additive effects may not be useful if the allelic frequencies in the reference population are different from those in the test population.

In this sense, it becomes evident that quantification of epistatic effects plays a fundamental role in genomic studies. Phillips [29] suggested that epistatic effects could be investigated over the full genome, i.e., take into account exhaustive pairwise combinations of genes. The biggest problem with this approach is related to the number of epistatic combinations when there are several candidate loci, i.e., considering *n* QTL, one can expect [*n(n - 1)/2*] epistatic combinations of first order without taking into account its interaction type (additive-additive, additive-dominant, dominant-additive and dominant-dominant). In other words, if the geneticist works with 100 QTL, it is necessary to exploit 4,950 interactions and to take into account all types of epistasis, this number rises to 20,000 allelic interactions. Currently, genome-wide studies have used a significant number of data points (usually 5 K to 1 M markers), which makes it difficult to exploit universal epistasis in multiple marker models.

Although methods to estimate major effects of QTL or markers in oversaturated maps has increased in the last few years, models considering the inclusion of marker interactions in the genomic analysis are scarce [28, 39, 40]. Learning strategies have been applied for epistasis estimates in the context of big data, such as Machine Learning (ML) decision trees [4, 14], information theory [8, 25] and multifactor dimensionality reduction (MDR) [28]. In the statistical framework, mixed models based on likelihood inference have been used to estimate epistatic effects using animal models and epistatic G-BLUP based on genomic additive and dominant matrices. In these models, the pairwise epistasis effects are ignored, and only the genomic epistatic values are taken into account for genomic prediction [13, 22, 27, 35]. Other approaches have estimated epistasis using markers with high marginal effects, which cannot explore minor epistatic interactions and the universal epistasis hypothesis [16].

Another interesting approach to estimate epistasis was proposed by Ronnegard and Valdar [31] and Ronnegard and Lee [30] using double hierarchical generalized linear models (DHGLM) to capture vQTL. These vQTL can partially retrieve epistasis signals, but it is not sufficiently informative about which loci are involved in the interactions controlling the traits or what type of epistasis is present in each interaction.

Using Bayesian inference, Xu [38] and Xu and Jia [40] studied first-order epistasis in multiples markers and showed the power of empirical Bayes approach to identify QTL. However, the exhaustive pairwise combination proposed by these authors is feasible only by a small number of markers and may be computationally intractable in a genome-wide context. Because the actual statistical models can easily handle a high number of markers, the main challenge is to include epistatic interactions without strongly altering the model dimension. An alternative is the use of Bayesian strategies, which enable us to identify epistatic interactions without severely changing the model size. In other words, the model dimension could be modified during the Monte Carlo Markov Chain process (MCMC) rather than considering all epistatic interactions in a final oversaturated model. In this scenario, the model dimension can be increased or decreased in each sampling cycle by inserting or deleting epistasis using probability rules. In this context, the reversible-jump [10] method could be used as a powerful tool for epistasis scans in the genome-wide analysis, mainly, when the model dimension is truncated over the markers but the number of epistatic loci is allowed to vary in its space.

The reversible-jump Monte Carlo Markov Chain (RJMCMC) technique has been successfully applied in genome studies when the number of QTL is unknown [41, 43–45, 47]. However, none of these works use RJMCMC to exhaustively determine epistatic interactions. Thus, this work was proposed to examine the efficiency of reversible-jump when there are thousands of candidate epistases in the genome analysis using simulated and real datasets.

## Results

### Simulated study

**Fig. 1:**
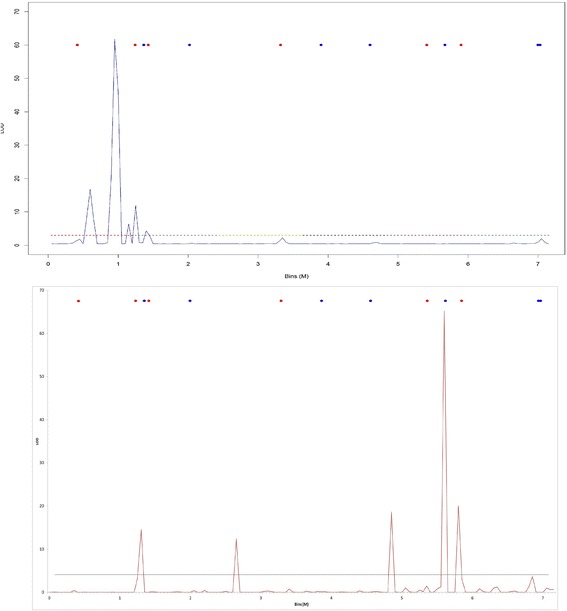
Genomic profile for QTL in seven simulated linkage groups using RJMCMC (*upper graph*) and empirical Bayes (*lower graph*). *Red dots* represent the non-epistatic QTL, and *blue dots* represent the simulated epistatic QTL. The dotted line represents the LOD criterion (LOD = 3)

The results related to the simulated study revealed that our model presents a good ability to find QTL with genetic additive and dominant effects (Fig. 1). In this study, the QTL were named according to the marker interval where they were identified or simulated. Two out of six principal QTL were found on the exact simulated position (second and third red dots); one was found at 19 cM away from the simulated QTL (the first red dot), and one marker presented a non-significant peak. Actually, the LOD = 2.89 at the fourth red dot. Our analysis did not find two out of the six simulated QTL in linkage groups 5 and 6. One epistatic QTL was identified presenting as a principal effect; in other words, this QTL was simulated presenting only epistatic effects and null for additive and dominant ones. Thus, the false discovery rate (FDR) in our study for QTL could be considered 1 out of 104 because the single peak showing one false discovery for principal QTL was actually an epistatic one. The same pattern was observed for the epistatic QTL localized at the intervals 91 and 94, but the observed peak was not significant. The strong shrinkage effect observed in our method can be verified by the number of peaks (5) in a total of 104 simulated markers (Fig. 1). The larger peak shown in this figure could be considered a spurious QTL because none of the simulated QTL were simulated in that position.

When the simulated example was analyzed by the PROC QTL program based on empirical Bayes, the results were very different from our methodology. Although both methods found some simulated QTL, the empirical Bayes captured more epistatic QTL in the genomic profile than that of non-epistatic QTL, presenting two false positives. Our method presented the opposite result, highlighting non-epistatic QTL in the genome profile. On the other hand, the empirical Bayes showed a highlighted peak in a true QTL, whereas our method was in a false positive. Thus, both methods showed different genomic profiles, but RJMCMC was more efficient in the control of false positives.

**Fig. 2:**
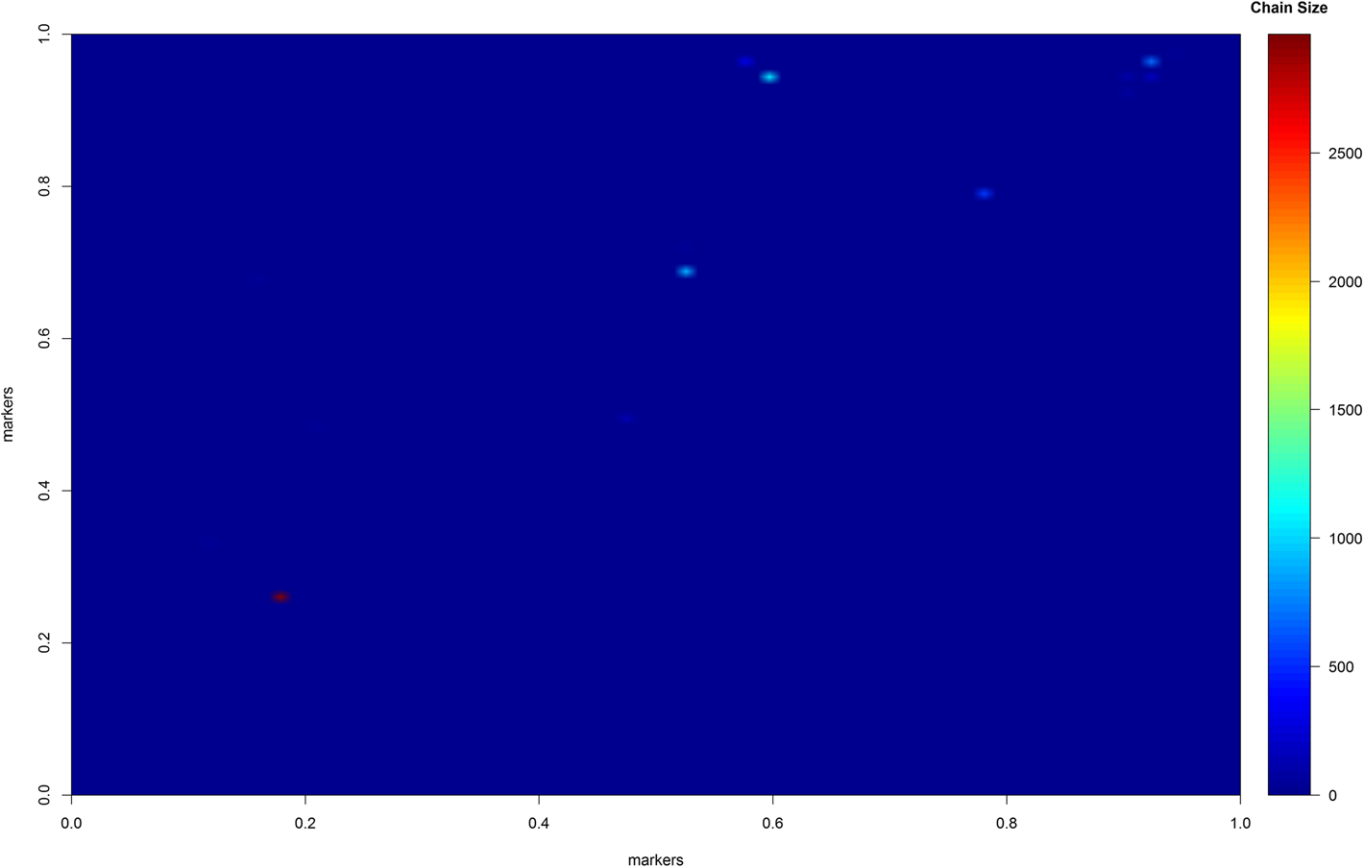
Heat map for epistatic QTL and chain size in the reversible jumping process. The more *red* the epistatic intensity is, the more time the QTL was retained in the MCMC process, meaning its effect on the epistatic complex is more likely

In Fig. 2, one can observe the heat-map for the length of the RJMCMC chain in epistatic QTL. All epistatic interactions were visited at least once, but one QTL presented a prominent chain size (the highlighted red point). In other words, this QTL was included during the MCMC process, and practically, it was not deleted in further MCMC sampling. This pair of QTL, named 18x26, was very close to true QTL, names here named as 19x25; the QTL peaks were 8.2 and 3.4 cM away from QTL 19 and 25, respectively. The other five green dots represent the QTL with larger chains when compared to other candidates.

**Fig. 3:**
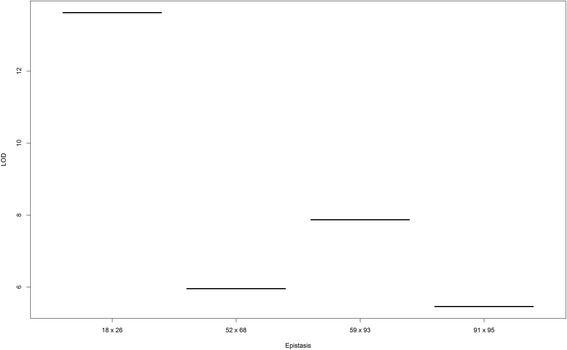
Selected epistatic effects based on chain length (>400). The simulated QTL were 19x25, 54x72, 59x91 and 59x94

To summarize the heat-map, we selected the epistases presenting final chain length up to 400 and found that four of these epistases presented LOD > 5 (Fig. 3). Furthermore, only one spurious epistasis was found, but the marker interval representing this false epistasis (91x95) was practically that determined individually for other true simulated epistases (59x91 and 59x94). In this figure, the estimate of epistasis, which was closer to the true simulated epistases (59x91 and 59x94) was that represented by the combination interval 59x93, presenting a genetic distance of 0.3 and 13.6 cM from simulated 59x91, respectively, and 1.2 and 5.96 cM from 59x94, respectively. Furthermore, estimated epistasis 52x68 was very close to the simulated 54x72, presenting a distance of 10.5 and 6.05 cM.

**Fig. 4:**
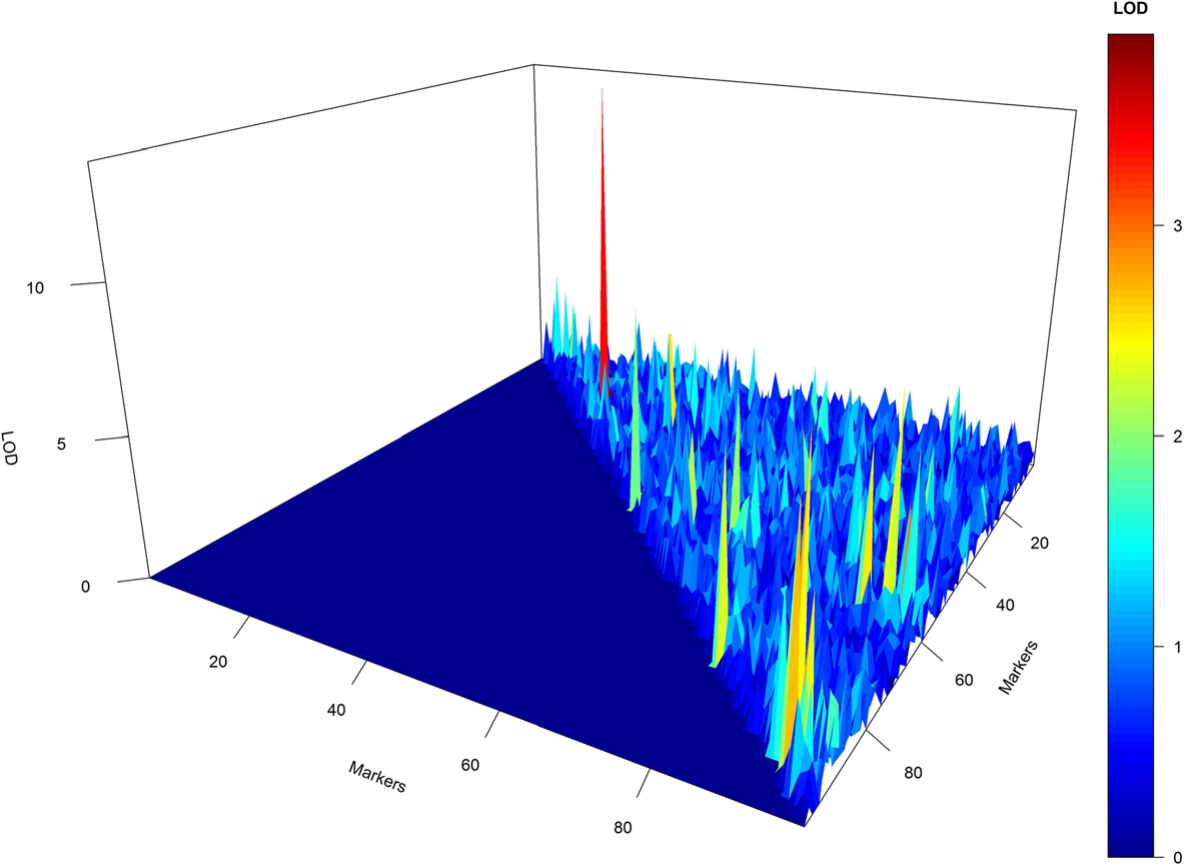
3D plot for raw epistatic effects of each QTL in the simulated study

**Fig. 5:**
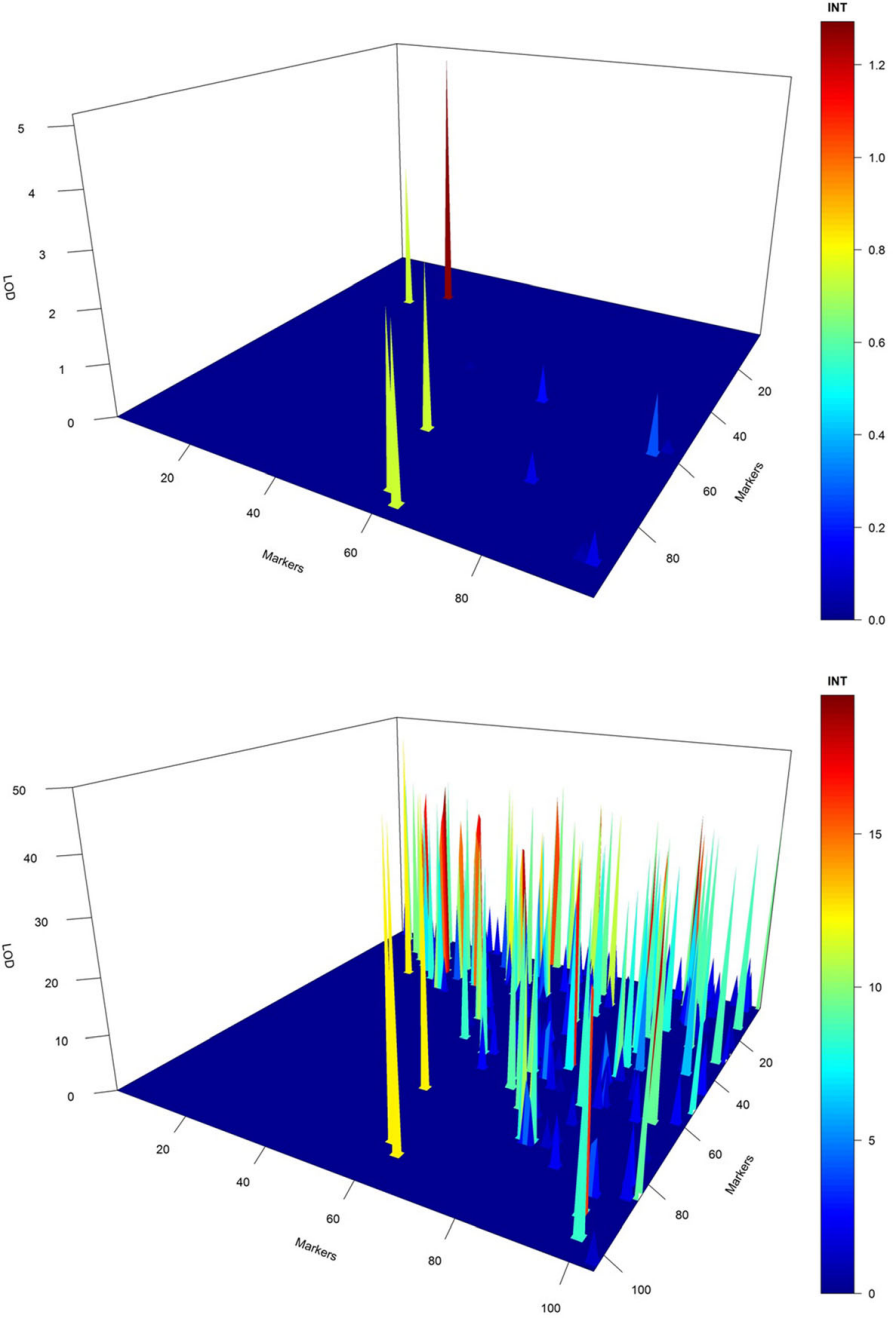
3D plot resolution for weighted epistatic effects obtained by RJMCMC (*upper graph*) and empirical Bayes (*lower graph*). The *green* peak on the *left side* of the 3D plot represents the simulated epistatic effect, and right size represents the estimated epistatic effect

Figure 4 represents the raw 3D plot for all retained epistases. In this figure, it is possible to observe that all epistatic interactions were visited at least once. However, this graph is not reliable because the epistases were compared only with their LOD magnitude and not by their constancy in the model. To avoid false epistasis discovery, we used a 3D plot weighted by the length of the chain. Using the weighted LOD, we obtained a high-resolution graph (Fig. 5). In this figure, the symmetry is evident between the simulated epistasis 19x25, 54x72, 59x91 and 59x94 and the estimated one 18x26, 56x68, and 59x93. However, “ghost” epistases were also observed (76x84). Overall, by the results from the simulated data, we can infer that our method was satisfactory for the identification of major QTL and presented a real ability to identify epistasis.

On the other hand, the Xu [38] and Xu and Jia [40] method was not effective for finding true epistasis. Several false positives were found, although no false negatives were observed (Fig. 5).

### Real maize dataset

**Fig. 6:**
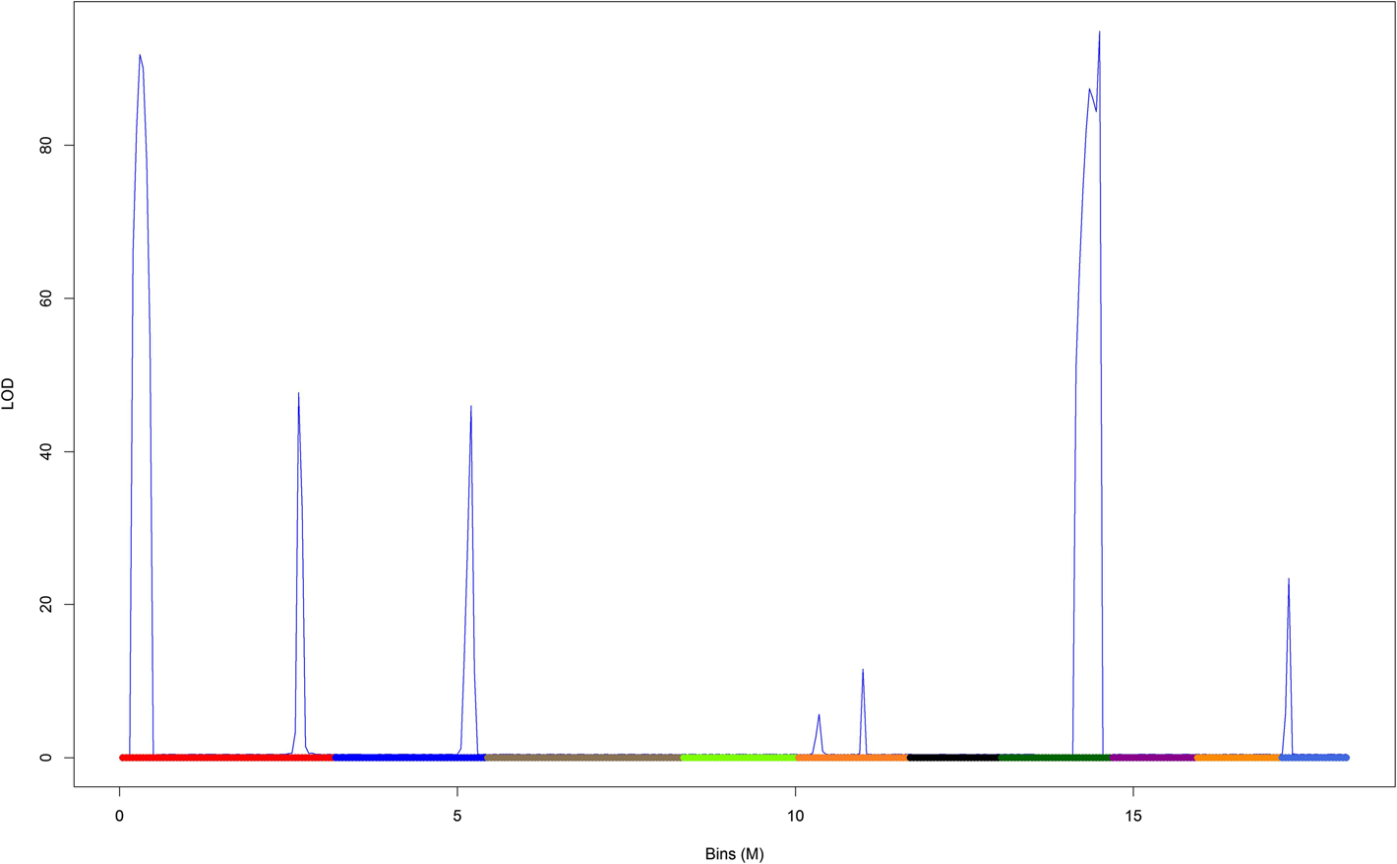
Genomic profile for QTL across the 10 linkage groups for grain yield in real maize data. The LOD score criterion was LOD = 3

In the real maize dataset, eight QTL of larger principal effect were found (Fig. 6). These QTL are distributed across five linkage groups and, in general, presented higher dominance effects over the additive ones (Table 1). It is evident that the dominance effects were superior to the additives ones, indicating a possible effect of heterosis acting on the grain yield in maize. Although the number of major QTL was relatively small, the number of epistatic QTL found was slightly higher (ten QTL acting in five epistatic combinations) (Table 2). Again, the dominance effects were highlighted in the epistasis interactions, and it becomes more evident when we observe the magnitude of dominant-dominant epistasis acting in the control of this trait. Most of the epistatic interactions were concentrated in the 1^st^, 2^nd^, and 5^th^ linkage groups. The 8^th^ linkage group also presented a high QTL epistatic effect related to additive-dominant and additive-additive interactions.

**Table 1:** Principal QTL effect with LOD > 3 for grain yield in real maize data

QTL	Interval	d	Bin	*a*	*d*
1	umc1177/bnlg1178	0.30	1.00–1.01	0.00	0.71
19	bnlg1720/umc1737	2.65	1.10	−0.01	0.10
39	umc1042/ umc1560	2.06	2.07–2.08	0.00	0.12
72	bnlg105/umc1879	0.37	5.03	0.00	0.56
77	mmc0282/umc1524	1.01	5.05–5.06	0.18	−0.68
94	phi034-bnlg657	1.38	7.02	0.00	0.70
95	bnlg657-umc1112	1.52	7.02−7.03	0.00	0.84
120	umc1576-bnlg1716	0.17	10.02–10.03	0.00	−0.06

d Genetic distance from the first marker, *a* additive effect, *d* dominant effect

**Table 2:** Decomposing the epistasis for grain yield in the real maize dataset with epistatic markers selected based on LOD criterion (LOD > 3)

Epistasis	*Q*_*i*_		*Q*_*j*_					
	Interval 1	Bin1	Interval 2	Bin2	*aa*	*ad*	*da*	*dd*
6×25	bnlg439/bnlg1203	1.03	umc1227/mmc0111	2.01	0.025	0.055	0.000	0.120
7×70	bnlg1203/bnlg1917	1.03–1.04	umc1365/bnlg1660	5.01–5.03	0.005	0.024	−0.001	0.042
9×11	umc1558/bnlg2295	1.04	umc1811/bnlg2025	1.04–1.05	0.008	−0.005	0.032	0.457
19×70	bnlg1720/umc1737	1.10	umc1365/bnlg1660	5.01–5.03	0.002	−0.038	0.007	0.125
70×105	umc1365/bnlg1660	5.01–5.03	bnlg1056/phi080	8.08	−0.057	−0.076	0.015	0.013

*aa*, *ad*, *da* and *dd* represent additive-additive, additive-dominant, dominant-additive and dominant- dominant epistatic effects, respectively; *Q*_*i*_ represents the first QTL, and Qj represents the second QTL

In Fig. 6, one can observe eight main QTL peaks. Clearly, not all linkage groups showed any major or epistatic QTL. In other words, in this study, linkage groups 3, 4, 6 and 10 showed no QTL.

**Fig. 7:**
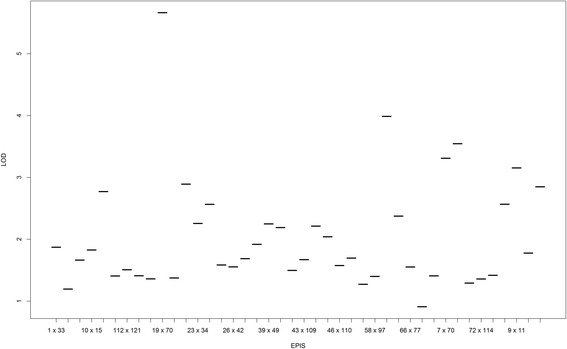
Selected epistatic effects based on chain length for grain yield in real maize data (>400)

Several epistatic QTL presented constancy in the model during the RJMCMC process. However, contrary to the LOD scores observed in the simulation study, the LOD scores for these QTL were very low. Thus, among all epistatic effects found in our model applied to the real dataset, only five epistases had a chain length larger than 400 and an LOD score greater than 3. No QTL with smaller chain size presented LOD scores greater than 3 (Fig. 7).

**Fig. 8:**
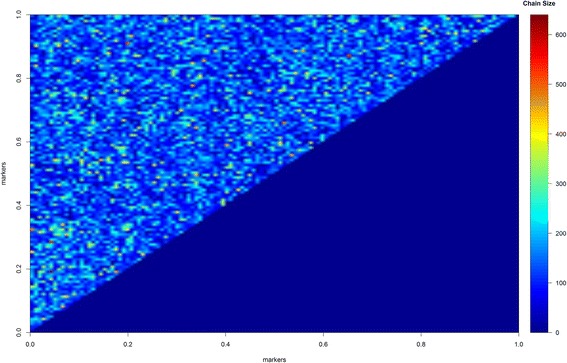
Heat map for epistatic QTL and chain size in the reversible jumping process for grain yield trait in real maize data. The redder the epistatic intensity is, the more time the QTL was retained in the MCMC process, meaning its participation in the epistatic complex is more likely

In Fig. 8, one can observe the full picture regarding the length of chains in the RJMCMC process. A different scenario was noted from the simulated study concerning the chain length. In other words, in the simulated study, we observe few epistatic QTL presenting a long chain and a small number of red dots in the heat map. In the real dataset, on the other hand, the heat map showed several hot spots, indicating that various epistatic QTL of lower effect may act in the genetic control of maize grain yield. For example, the significant QTL presented dominant-by-dominant epistatic variance equal to 0.028, and the non-significant epistatic variance was 0.010. The additive variance was, by magnitude, only 30% of this amount (0.003). This magnitude may be compared to other variance components. For example, the dominance variance in this study was 0.196, and epistatic additive-additive, additive-dominant and dominant-additive were 0.001, 0.002 and 0.000, respectively. The total genetic variance was 0.2395, and the residual variance was 0.068.

**Fig. 9:**
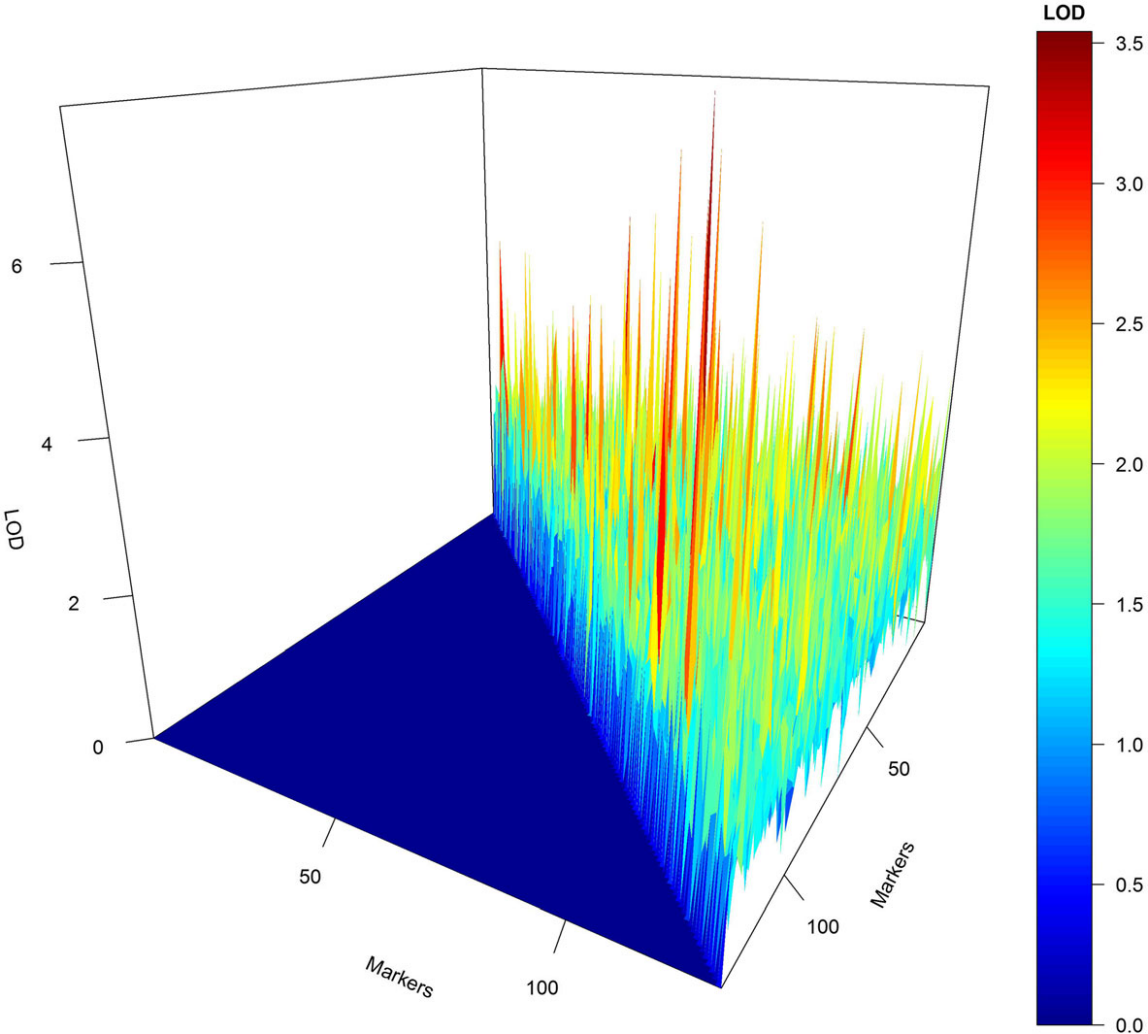
3D plot for raw LOD epistatic effects for the grain yield in the maize data set

**Fig. 10:**
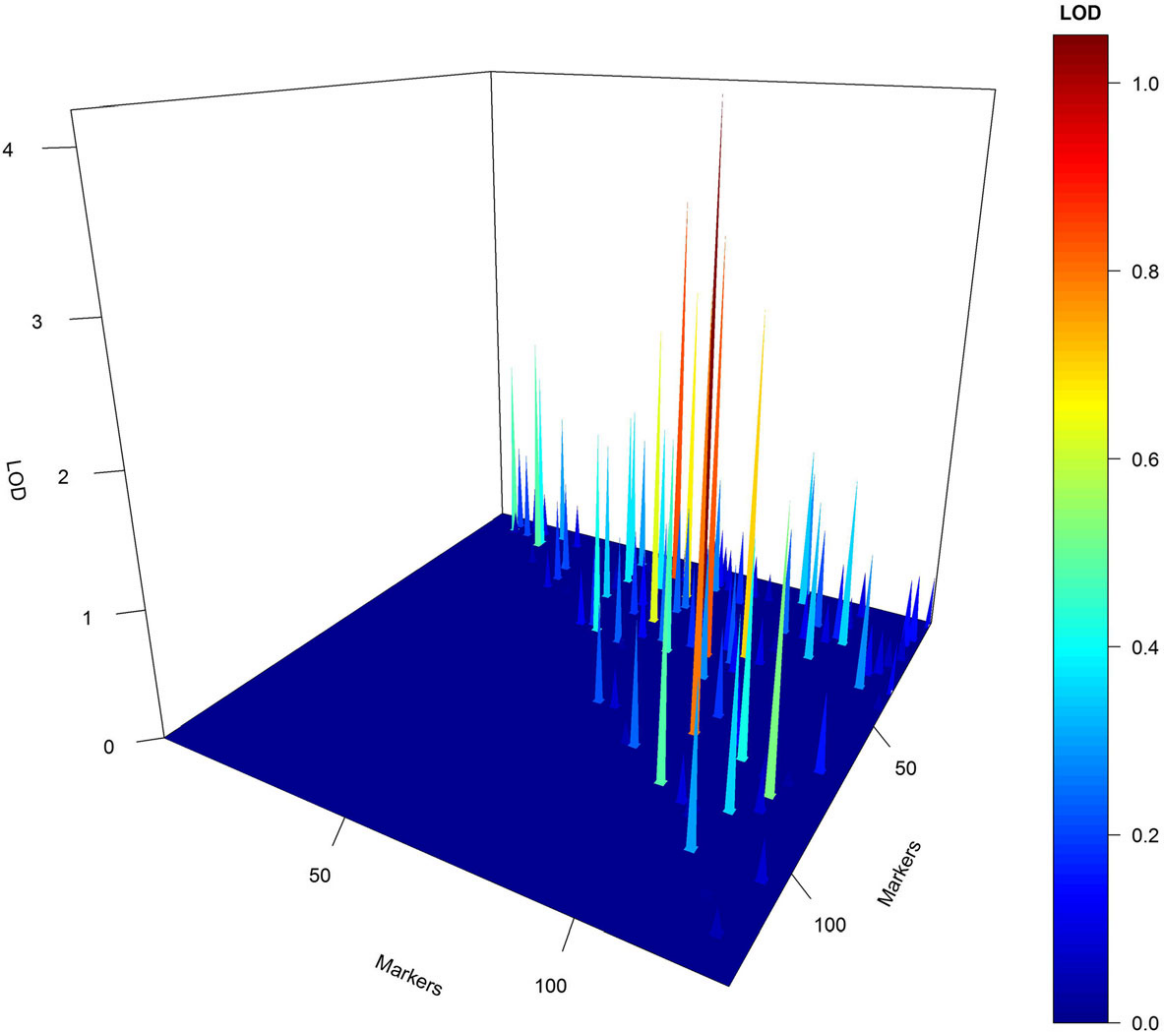
3D plot resolution for weighted LOD epistatic effects for the grain yield in the maize data set

More than five epistatic combinations presented LOD > 3. Fourteen epistatic interactions and 19 different QTL were related to these orange/red peaks. However, only five interactions presented a chain length >400. In this figure, it is evident that all epistatic combinations were visited and incorporated at least once into the model during the RJMCMC process (Fig. 9). In the weighted 3D plot (Fig. 10), it is possible to observe five high interactions based on their red/orange peaks. Differing from that observed in the simulated dataset, the shrinkage effect related to maize data was not as intense as that observed in simulated data; because of this, several minor epistatic effects could be observed in this plot. Additionally, the same epistatic interactions previously identified as promising (Figs. 7, 8, 9, and 10) were also established in this plot (highest peaks).

**Fig. 11:**
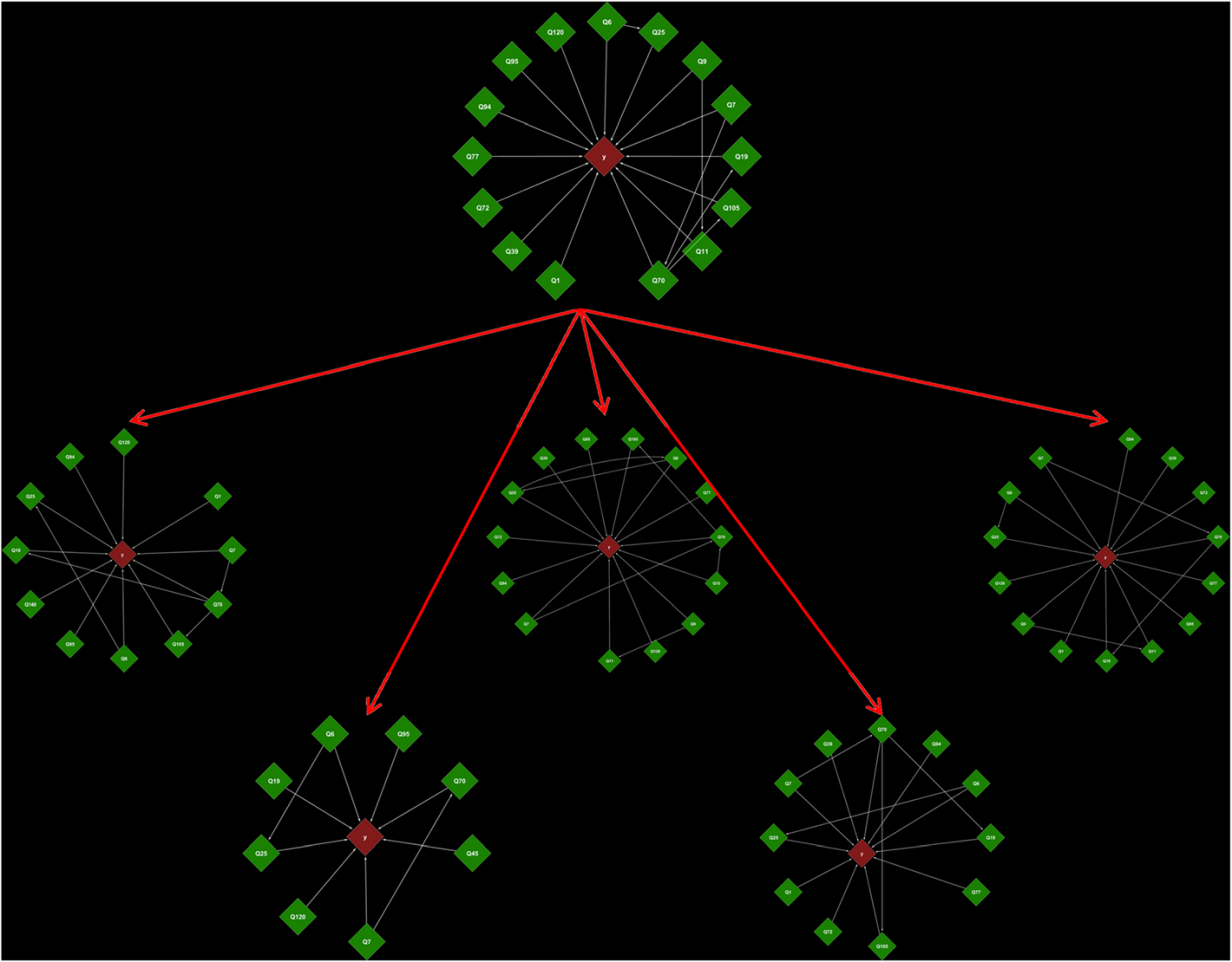
Complex epistatic network for grain yield (y) in maize considering principal and epistatic QTL (Q). The top principal graph was built by the full dataset, and the five sub-graphs were built by 5-fold analysis

Figure 11 presents the final estimate of the genetic architecture and the results of the 5-fold analysis. Obviously, this naïve network does not represent the real scenario of the genetic architecture for maize grain yield. However, it is useful for observing the QTL presenting high importance and their relation to other significant QTL. For example, QTL 70 is related to three different epistatic interactions, and although it has no significant major effect (additive and dominance), its importance for grain yield is evident because it appeared in all cross-validation analyses. The only QTL showing relevant epistatic and high main effects was “QTL 19”, localized in linkage group 1, but this QTL did not appear in all cross-validation scenarios.

## Discussion

The main challenge in genome-wide models is dealing with high dimension matrices related to marker information. In other words, if *n* is related to the number of phenotyped individuals and *p* is related to the number of markers, it is evident that, if *p* > = *n*_,_ a simple regression analysis is restrictive to estimate marker effects. The inclusion of two-way epistasis makes *p* ≫ *n*_,_ and the analysis becomes prohibitive by classical least square methods and computationally intractable by multiple markers approaches. In our simulation study, even using only 104 markers, the number of additive and dominant effects in regression models was 194 in each interval (each linkage group present *k -* 1 intervals). Furthermore, for each type of epistasis, there are 4,656 possible interactions, and using all combinations, i.e., additive-additive, additive-dominance, dominance-additive and dominance-dominance, there are a total of 18,624 epistases plus 194 main effects, totaling 18,818 estimates. For the real dataset, we explore 33,024 epistatic effects plus 258 additive and dominant.

Because this space is not so high in our models, it was possible to visit all candidate interactions and select marker intervals very close to the simulated epistatic QTL. In addition, the maximal model dimension observed during the MCMC process corresponded to 226 effects, that is, the highest number of epistatic interactions running simultaneously on the model was equal to eight, allowing a high computational efficiency, presenting a computational difference from the model without epistasis for only 360 s.

In the simulation study, the power of our analysis to find the main QTL was satisfactory in the sense that our model had overlooked only two out six simulated principal QTL (two false negatives). An interesting result is the presence of peaks in the genomic profile where the simulated epistatic QTL did not present any principal effect. This type of “cloud effect” is discussed extensively by Carlborg and Haley [5], and it is defined as “co-adaptative epistasis”, i.e., the pairs of epistatic markers present some biological advantage when the loci are in homozygosis, although the individual markers do not present a significant effect *per se*. This QTL (19) localized on chromosome 2 was not simulated with this intent. However, because we simulated high negative dominant epistasis and low additive-additive, when the individuals were homozygous, it presented some advantages for this locus, and for this reason, this locus was identified as a “true” principal QTL. This “cloud effect” was observed (although not as evident) for other epistatic QTL across the genomic profile.

The success of the analysis could be evaluated by the length of the chains for the simulated epistatic QTL, i.e., whether the true QTL are retained in the model during the MCMC process. In our study, the simulated QTL were well represented by their neighboring markers, which in turn presented a long chain compared to other candidate intervals. The only spurious epistatic interaction (91x95) actually captured the simulated epistatic QTL 91 and 94. This “ghost effect” is expected because these markers are very close each other; actually, the average distance between these markers is approximately 7.8 cM. These also explain the significant epistasis 59x93 instead of the simulated ones 59x91 and 59x94.

As mentioned above, the raw LOD score presents a low resolution to identify the epistatic pattern. It may occur when the pairwise QTL effects present a high LOD score but, on the other hand, a small chain and a poor mixture in the posterior. Thus, high values of LOD could exhibit a leverage effect on the LOD means. In this regard, our *ad hoc* approach was effective for cleaning spurious epistatic effects on the model, achieving a better resolution in the LOD plot. Furthermore, this “trick” presents a special concordance with the results presented in Figs. 3 and 7. Consequently, we recommended the two stage plot, that is, to first use the truncated graph based on chain size (Fig. 3) and further select markers presenting with LOD above 3 instead of using the LOD score and further weighting these LOD by chain size.

The empirical Bayes approach proposed by Xu [38] was utilized for comparison because it is one of the few that performs exhaustive searching in the epistatic space and estimates all epistasis combinations (additive-additive, additive-dominant, dominant-additive and dominant-dominant). Although empirical Bayes and RJMCMC present similar proposals, our method performs particularly better than Xu’s [38] method for searching epistatic effects. The differences in power and false positive rate in major QTL analyses were marginal between these two approaches. However, the superiority of our technique for finding true epistasis using the LOD over the chain size is evident. Both methods found several false peaks in the raw analysis, but given that in RJMCMC the size of the chain is determinant and can be used as probabilistic criterion to select epistasis, a high-resolution analysis could be obtained (the larger the chain size, the more likely the permanence of the epistasis in the model).

Other methods could also be used to compare with RJMCMC, but most of them do not follow our proposal, that is, they do not estimate all candidate epistases and/or do not operate effectively in the interaction space. If so, they need to use *ad hoc* procedures, such as multi-stage analysis, by screening major markers [16].

Although the Xu [38] method is very attractive to the user because it is available in a friendly SAS macro and any programmer with basic knowledge of SAS can easily operate the program, it is very restrictive when several markers are included in the analysis. For example, when we used 400 markers in our simulation, the program did not work properly, and it was not possible to estimate the 319,000 epistases. For this reason, it was not possible to compare it with our method, which worked very well in this scenario. Therefore, all results related to this simulation were inserted as supplemental material (Additional file 1: Figures Sl; Additional file 2: Figures S2 Additional file 3: Figures S3 Additional file 4: Figures S4 and Additional file 5: Tables S1; Additional file 6: Tables S2). Some observations can be drawn from this simulation scenario with 400 markers: 1) most of the simulated epistases were found with low genetic distance from the simulated ones, but the sizes of the chains were not safely high enough to make inferences because it presented as a poor mixture during MCMC (Additional file 5: Tables S1 and Additional file 6: Tables S2); 2) using 100,000 iterations, it was possible to explore 86% of the 319,000 possible epistases, which shows that the number of sampling in RJMCMC can be a limiting factor (Additional file 2: Figures S2); 3) among the epistases selected by the same aforementioned criteria, some had low genetic distance from the simulated epistasis (Additional file 5: Table S1); 4) the model was very effective for finding the principal QTL (additive and dominant). Therefore, these results show that it is possible to estimate major and epistatic effects in a one stage model, but some precautions are necessary to regulate the number of iterations in RJMCMC.

An issue of our method that must be discussed refers to the inclusion or deletion of full epistatic terms in the decision rules (24 and 25) instead of the isolated epistatic effects in the model. We think sampling isolated effects may be less efficient for RJMCMC because some QTL can be true epistatic, with at least one epistatic effect as true, but present a null effect for an isolated interaction. In this scenario, more rounds of MCMC could be necessary. In addition, given that the whole epistatic effect in the pair of QTL presents a Bayes factor of less than one, there is no reason to test the isolated effects.

In the real dataset, although LOD scores selected only five epistasis effects, some epistases presenting minor importance were kept in the model. This result confirms the complexity involving the epistasis related to grain yield in maize and the difficulty of finding pairwise markers controlling this trait [9]. For example, the most important marker interval found in our work was related to QTL 19 and QTL 70. QTL 19 is localized inside the marker interval bnlg1720-umc1737 localized on linkage group 1. This marker is localized on chromosome 1 in the maize genome, and its interval ranges from 274,684,822 to 290,556,990 bp (http://www.maizegdb.org/data_center/locus/144977 and http://www.maizegdb.org/data_center/locus/292557). Several QTL have been reported in this bin. For example, Frascaroli et al. [9] found some epistatic effects among intervals including the marker bnlg1720.

The more relevant epistatic interval was related to umc1365-bnlg1660. Although this result could be considered as encouraging, it is evident that the genomic window between these markers is vast, ranging from bins 5.01 to 5.03 and presenting approximately 34 cM of distance [19]. The genetic distance between the found QTL and the umc1365 marker was approximately 4.3 cM; using a naïve approach, it corresponds to a physical distance of 1.3 Mpb [19]. Therefore, the corresponding region for this QTL in chromosome 5 is approximately 6.18 Mpb. It is worth highlighting that, between the regions 6.105 and 6.18 Mbp, several genes are found to be expressed in different cycles of maize crops [33] (http://www.maizegdb.org/gbrowse/maize_v2/?name = Chr5:5,377,553..5,473,955;a = Chr5 ± LOCUS_LOOKUP ± umc1365 ± 5377553..5473955;style = LOCUS_LOOKUP ± glyph = span ± fgcolor = green ± height = 30 ± description = %22The%20estimated%20region%20for%20umc1365%20based%20on%20locus%20lookup%22 ± hilite = yellow).

The k-fold analysis of the real dataset revealed what was expected, that is, that the RJMCMC method finds a local maximum in the epistatic space, which becomes evident when we look at the different interrelations among the QTL and phenotypes across the CV analysis (Fig. 11). However, an encouraging result is that the most important QTL were very stable over the unbalanced process.

The “pervasive epistasis” hypothesis suggested by Mackay [20, 21] may explain the result observed in Figs. 8 and 9 and also the importance of epistatic effects on the genetic variance in this study. When pervasive epistasis is present on a quantitative trait, Mackay notes the so-called missing heritability acting on the trait, hampering the genetic progress in artificial selection [48]. Thus, using a model that simultaneously includes additive, dominant and epistatic effects on all pairwise markers, geneticists can exploit the genetic architecture to avoid poor genomic predictions.

The searching strategy for epistasis has been proposed by several authors in genome-wide studies to incorporate its effects into the model [16, 28, 31, 32, 38, 40]. However, several of these methods are based on undirected epistasis estimates for multistage strategies; in these circumstances, the genetic architecture may not be correctly depicted. Xu [38] and Xu and Jia [40] proposed an epistatic model where additive marker effects and epistasis are incorporated into the model. In a total of 121 and 127 markers used by these authors, the Xu method recovered only 7,260 and 8,001 pairs of epistatic interactions, respectively. Our method presents some similarities to the Xu approach in the sense that all pairwise epistasis interactions are estimated. However, our model may be more desirable because all pairs of epistasis may be visited without significantly increasing the model. The method applied here could be extended to other branches of genomics. For example, in microarray models, independence is assumed among the genes across the data, which forces a sparse matrix to facilitate estimates of case–control contrasts. One strategy would be to apply RJMCMC in the sparse covariance matrix relaxing to an assumption of independence among genes.

The main difficulty related to our analysis was separating the principal and epistatic QTL in a marker interval; specifically, the QTL position in the epistasis marker interval was not always the same as that of the principal one. For example, for QTL 19, the main effect was 4.8 cM away from umc1365, and the epistatic one was 4.3 cM away from this marker. This difficulty may be overcome with an oversaturated genome as obtained in genome-wide studies; in other words, using the markers *per se* instead of the marker intervals. In this situation, the epistasis might be obtained by pairwise markers, and the problem of QTL position could be avoided. However, as more markers are inserted into the model, the length of the MCMC chain necessary to visit all pairs of epistasis becomes enormous and, in this case, markers bins may be requested for epistasis estimates [39].

## Conclusions

Our work provides useful results related to epistasis studies using statistical models. It is evident that, in a saturated genomic framework (where there are thousands of markers), the results could be more practical, for example, in the genome-wide selection or GWAS context. However, because the QTL searching models are more sophisticated than genome-wide regression models, a slight change in our approach could enable it to be applied in genome-wide association studies or genome scanning for QTL in structured populations.

## Methods

### Statistical genetic model

Genome-wide searching requires that several candidate regions be mapped simultaneously. In this context, the multiple marker interval methods are very attractive because all minor and major QTL are considered jointly in the model to recover the genetic variance. Considering all unobserved QTL and their possible interactions at the same time, the theoretical linear model, including all first-order epistasis for QTL mapping, is given by

1 $$ {y}_i=\mu +{\displaystyle \sum_{k=1}^p{x}_{ik}{a}_k+{\displaystyle \sum_{k=1}^p{w}_{ik}{d}_k+{\displaystyle \sum_{k^{\prime}\ne k}^t{\eta}_{ik\prime }a{a}_{kk\prime }}}}+{\displaystyle \sum_{k^{\prime}\ne k}^t{\gamma}_{ik\prime }a{d}_{kk\prime }}+{\displaystyle \sum_{k^{\prime}\ne k}^t{\varpi}_{ik\prime }d{a}_{kk\prime }}+{\displaystyle \sum_{k^{\prime}\ne k}^t{\varphi}_{ik\prime }d{d}_{kk\prime }}+{e}_i $$

where *y*_*j*_ is the phenotypic value at the *j*th individual, *μ* is the vector of the general mean, *a*_*k*_ and *d*_*k*_ are the additive and dominant effects of the k^th^ QTL, respectively, and *aa*_*kk´*_, *ad*_*kk´*_, *da*_*kk´*_, and *dd*_*kk´*_ are related to additive-additive, additive-dominant, dominant-additive and dominant-dominant epistatic effects, respectively, for loci k and k`. The residual is assumed as Gaussian distributed with a mean of 0 and variance equal to *σ* ^2^. The unobserved QTL variables *x*_*ik*_ and *w*_*ik*_ were assumed under F_2_ metric, presenting orthogonal contrasts for all effects [23, 42, 46]. Thus, it was assumed that

2 $$ {x}_{ik}=\left\{\begin{array}{ccc}\hfill 1\hfill & \hfill if\hfill & \hfill QQ\hfill \\ {}\hfill 0\hfill & \hfill if\hfill & \hfill Qq\hfill \\ {}\hfill -1\hfill & \hfill if\hfill & \hfill qq\hfill \end{array}\right.\kern0.5em \mathrm{and}\kern0.5em {w}_{ik}=\left\{\begin{array}{ccc}\hfill -1/2\hfill & \hfill if\hfill & \hfill QQ\hfill \\ {}\hfill 1/2\hfill & \hfill if\hfill & \hfill Qq\hfill \\ {}\hfill -1/2\hfill & \hfill if\hfill & \hfill qq\hfill \end{array}\right. $$

It is evident that the random variables *x*_*ik*_ and *w*_ik_ are unobserved, which can be inferred from neighboring markers and their relative positions in the genome. Considering the orthogonal model, the epistatic variables *η*_*ik´*_, *γ*_*ik´*_, *ϖ*_*ik´*_ and *φ*_*ik´*_ are obtained by the element-wise product of the additive and dominant matrices, i.e., they were built by dot product given by (*x*_*ik*_, *w*_*ik*_) ∘ (*x*_*ik´*_, *w*_*ik´*_) [23, 42, 46]. The model (1) takes into account all epistatic interactions and, as mentioned above, might become overparameterized given the small number of markers used for QTL mapping. Thus, it is necessary to set up an algorithm that starts with an additive-dominant model and further makes insertions or deletions of epistatic effects across the model.

Under the overparameterized model, the *t*-index on the top of the summation may range from 0 to (p^2^ − *p*)/2. Because *t* is unknown, the size of the genetic model is missing information. However, *t* can be inferred by reversible-jump Monte Carlo Markov Chain (RJMCMC), building an epistatic model presenting with variable dimensions. The main idea of RJMCMC is to find a model in which the number of variables is unknown. Because a Poisson process can model the number of variables (*t*), RJMCMC uses this distribution plus the likelihood given *t* to decide the number of parameters in the model. However, given that the size model can increase to (p^2^ − *p*)/2 or even to lower dimension models that include spurious variables, the reversible process is necessary to arrive at a maximum local. For this, the Hastings correction becomes necessary. In this context, details of the full conditional and RJMCMC algorithm plus the decision rule to include or delete an epistatic effect in the model will be presented in next.

### Likelihood function

Assuming that the *t*-index (number of epistatic effects on the architecture of the trait) is an unknown variable, the likelihood might be described as below.

To simplify the statistical notation, we will consider the following vector correspondence: *y* = *y*_*i,*_ where {*i* = 1,2,3,…,*n*}. Additionally, we will also assume that *Ω*_*k*_ = [*η*_ik ´_, *γ*_ik_, *ϖ*_ik_, *φ*_ik_] and *Θ*_*k*_ = [*aa*_kk ´_, *ad*_kk ´_, *da*_kk ´_, *dd*_kk ´_]^*T*^. On the assumption of residual normal distribution, one can assume that the conditional distribution of y is given by

3 $$ p\left(y\Big|b,a,d,x,w,{\sigma}^2\right)=N\left(\mu +{\displaystyle \sum_{k=1}^p{x}_k{a}_k+{\displaystyle \sum_{k=1}^p{w}_k{d}_k+{\displaystyle \sum_{k=1}^t{\varOmega}_k{\varTheta}_k,{\sigma}^2}}}\right) $$

Alternatively, it can be presented in mathematical terms by

4 $$ p\left(y\Big|\lambda, b,a,d,\varTheta, x,w,\varOmega, {\sigma}^2\right)\propto {\left({\sigma}^2\right)}^{-n/2}\times \exp \left\{-\frac{1}{2{\sigma}^2}{\left(y-\mu -{\displaystyle \sum_{k=1}^p{x}_k{a}_k-{\displaystyle \sum_{k=1}^p{w}_{ij}{d}_j-{\displaystyle \sum_{k=1}^t{\varOmega}_k{\varTheta}_k}}}\right)}^2\right\} $$

The parameters of interest will be taken as *θ* = {*μ*, *a*, *d*, *Θ*_*k*_, *σ* ^2^}. For the classical Bayesian shrinkage model, the number of possible QTL is conditioned to the number of potential marker intervals [1]; consequently, for *m* markers in each linkage group, there are *m - 1* possible QTL. All of these QTL are retained in the model using multiple interval methods with a constant number *p* of markers [1]. However, the number of epistatic terms (*t*) is unknown and must be estimated by the data at hand.

### Prior distribution

Each parameter of the model (1) has a specific prior distribution. The general mean is assumed by Jeffrey’s prior given by *p*(*μ*) ∝ *k*. For additive, dominant and epistatic effects, normal prior distributions are given by $p\left({a}_k\right) \propto N\left(0,{\sigma}_{a_k}^2\right),\kern0.5em p\left({d}_k\right)\propto N\left(0,{\sigma}_{d_k}^2\right)$, $p\left(a{a}_k\right) \propto N\left(0,{\sigma}_{a{a}_k}^2\right),\ p\left(a{d}_k\right) \propto N\left(0,{\sigma}_{a{d}_k}^2\right),\ p\left(d{a}_k\right) \propto N\left(0,{\sigma}_{d{a}_k}^2\right),\ p\left(d{d}_k\right) \propto N\left(0,{\sigma}_{d{d}_k}^2\right)$. The specific variance for each effect presents prior information specified by $p\left({\sigma}_{a_k}^2\right)=p\left({\sigma}_{d_k}^2\right)=p\left({\sigma}_{a{a}_k}^2\right)=p\left({\sigma}_{a{d}_k}^2\right)=p\left({\sigma}_{d{a}_k}^2\right)=p\left({\sigma}_{d{d}_k}^2\right)\propto inv- scaled{\chi}^2\left(v=1,{s}^2=0\right)$. These priors have been described in detail by Xu [37].

Analytically, these priors result in improper marginal posterior distributions, as also verified by Ter Braak et al. [36] in Xu’s shrinkage model with specific variances. For dealing with this problem, the extended prior presented by Ter Braak et al. [36] was adopted, correcting the degrees of freedom to obtain a proper posterior distribution. Therefore, the prior distribution for the specific variance is as follows: Assuming *s* ^2^ = 0 and $v={n}_{\lambda_k}-1=-2\varDelta$, we have $p\left({\sigma}_{a_k}^2\right)\propto {\left({\sigma}_{a_k}^2\right)}^{\varDelta -1}$, where $\varDelta =-\frac{\left({n}_{a_k}-1\right)}{2}$, such that $0<{n}_{a_k}<1$ and, consequently, 0 < *Δ* < 1/2. Here, ${n}_{a_k}$ is an arbitrary value. In this study, we assumed ${n}_{a_k}=0.95$ from our previous experience with Bayesian shrinkage models ([1, 7]).

We have assumed the prior *p*(*λ*_*k*_) relative to the QTL position in the genome as uniform, specifically taking *M*_*k*_^*L*^ and *M*_*k*_^*R*^ as the bordering markers related to QTL *Q*_*k*_ *, L*_*k*_ and *U*_*k*_ as the distances between *M*_*k*_^*L*^ *↔Q*_*k*_ and *Q*_*k*_ *↔ M*_*k*_^*R*^; the uniform prior for each interval is given by a set of ordering numbers presenting equal probability ranging from *L*_k_ to *U*_k_. Thus, the joint prior distribution could be given by

5 $$ p\left(\theta \right)=p\left(\mu \right)p\left({\sigma}^2\right){\displaystyle \prod_{k=1}^pp\left({\lambda}_k\right)p\left({a}_k\right)p\left({d}_k\right)p\left({\sigma}_{a_k}^2\right)p\left({\sigma}_{d_k}^2\right)}{\displaystyle \prod_{k=1}^tp\left(a{a}_k\right)p\left(a{d}_k\right)p\left(d{a}_k\right)p\left(d{d}_k\right)p\left({\sigma}_{a{a}_k}^2\right)p\left({\sigma}_{a{d}_k}^2\right)p\left({\sigma}_{d{a}_k}^2\right)p\left({\sigma}_{d{d}_k}^2\right)} $$

In this prior, the *p-*index is given as known, and it is a function of the number of markers intervals used for mapping. However, the number of epistatic interactions (*t*) is unknown. Thus, we can assume that the number of epistatic interactions is modeled by a truncated Poisson distribution given by

6 $$ p\left(t\Big|\phi \right)\propto \frac{\phi^t{e}^{-\phi }}{t!} $$

where *ϕ* is the Poisson mean related to the number of epistases controlling the trait. In hierarchical modeling, the prior for *ϕ* can be assumed as a *gamma*(*τ* = 1, *υ* = 1)_,_ where *τ* is the shape parameter and *υ* is the rate parameter [39].

### Joint posterior distribution and full conditional distributions

On the basis of prior and likelihood distributions, the joint posterior distribution is given by

7 $$ p\left(\theta, x,w,\varOmega, t,\phi \Big|y\right)\propto p\left(y\Big|b,a,d,\varTheta, x,w,{\sigma}^2\right)p\left(x,w\Big|\lambda \right)p\left(\theta \right)p\left(t\Big|\phi \right)p\left(\phi \right) $$

Below, we present the full conditional for RJMCMC using Gibbs sampler for most of the posterior parameters instead the Metropolis–Hastings (M-H) algorithm as proposed in Sillanpää and Arjas [34]. The M-H algorithm will be used here only for sampling the QTL position.

#### Full conditional posterior for the reversible-jump algorithm

Given all priors and likelihood described previously, the conditional posterior distribution of the general mean can be obtained by

8 $$ \begin{array}{l}p\left(\mu \Big|\dots \right)=p\left(\mu \right)p\left(y\Big|b,a,d,\varTheta, x,w,{\sigma}^2\right)\\ {}p\left(\mu \Big|\dots \right)\propto {\operatorname{e}}^{{\frac{-n\left[\mu -\frac{1}{n}{\displaystyle \sum_{i=1}^{n_j}\left({y}_i-{\displaystyle \sum_{k=1}^p{x}_{ik}{a}_k-{\displaystyle \sum_{k=1}^p{w}_{ik}{d}_k-{\displaystyle \sum_{k=1}^t{\varOmega}_{ik}{\varTheta}_k}}}\right)}\right]}{2{\sigma}^2}}^2}\end{array} $$

Therefore, the overall mean can be sampled from a normal distribution presenting a mean equal to $\frac{1}{n}{\displaystyle \sum_{i=1}^{n_j}\left({y}_i-{\displaystyle \sum_{k=1}^p{x}_{ik}{a}_k-{\displaystyle \sum_{k=1}^p{w}_{ik}{d}_k-{\displaystyle \sum_{k=1}^t{\varOmega}_{ik}{\varTheta}_k}}}\right)}$ and variance of $\frac{\sigma^2}{n}$, where *p*( |....) means conditional on others parameters.

The conditional posterior for the kth additive effect can be given by

9 $$ \begin{array}{l}p\left({a}_k\Big|\dots \right)=p\left({a}_k\right)p\left(y\Big|b,a,d,\varTheta, x,w,{\sigma}^2\right)\\ {}p\left({a}_k\Big|\dots \right)\propto {e}^{-0.5{\sigma}^{-2}{\left[{a}_k-{\left({\displaystyle \sum_{i=1}^n{x}_{ik}^2+{\sigma}_{ak}^{-2}{\sigma}^2}\right)}^{-1}{\displaystyle \sum_{i=1}^n{x}_{ik}}\left({y}_i-\mu -{\displaystyle \sum_{k\acute{\mkern6mu}\ne k}^p{x}_{ik\acute{\mkern6mu}}{a}_{k\acute{\mkern6mu}}-{\displaystyle \sum_{k=1}^p{w}_{ik}{d}_k}-{\displaystyle \sum_{k=1}^t{\varOmega}_{ik}{\varTheta}_k}}\right)\right]}^T\left({\displaystyle \sum_{i=1}^n{x}_{ik}^2+{\sigma}_{ak}^{-2}{\sigma}^2}\right)\left[{a}_k-{\left({\displaystyle \sum_{i=1}^n{x}_{ik}^2+{\sigma}_{ak}^{-2}{\sigma}^2}\right)}^{-1}{\displaystyle \sum_{i=1}^n{x}_{ik}}\left({y}_i-\mu -{\displaystyle \sum_{k\acute{\mkern6mu}\ne k}^p{x}_{ik\acute{\mkern6mu}}{a}_{k\acute{\mkern6mu}}-{\displaystyle \sum_{k=1}^p{w}_{ik}{d}_k}-{\displaystyle \sum_{k=1}^t{\varOmega}_{ik}{\varTheta}_k}}\right)\right]}\end{array} $$

Thus, the posterior distribution for the additive effect is normally distributed, presenting a mean equal to ${\left({\displaystyle \sum_{i=1}^n{x}_{ik}^2+{\sigma}_{ak}^{-2}{\sigma}^2}\right)}^{-1}{\displaystyle \sum_{i=1}^n{x}_{ik}}\left({y}_i-\mu -{\displaystyle \sum_{k^{\prime}\ne k}^p{x}_{ik\prime }{a}_{k\prime }-{\displaystyle \sum_{k=1}^p{w}_{ik}{d}_k}-{\displaystyle \sum_{k=1}^t{\varOmega}_{ik}{\varTheta}_k}}\right)$ and variance equal to ${\left({\displaystyle \sum_{i=1}^n{x}_{ik}^2+{\sigma}_{ak}^{-2}{\sigma}^2}\right)}^{-1}{\sigma}^2$. For the dominance effect, the same normal posterior distribution is obtained, but presenting a mean equal to ${\left({\displaystyle \sum_{i=1}^n{w}_{ik}^2+{\sigma}_{dk}^{-2}{\sigma}^2}\right)}^{-1}{\displaystyle \sum_{i=1}^n{w}_{ik}}\left({y}_i-\mu -{\displaystyle \sum_{k=1}^p{x}_{ik}{a}_k-{\displaystyle \sum_{k^{\prime}\ne k}^p{w}_{ik\prime }{d}_{k\prime }-{\displaystyle \sum_{k=1}^t{\varOmega}_{ik}{\varTheta}_k}}}\right)$ and variance equal to

10 $$ p\left({d}_k\Big|\dots \right)=N\left[{\left({\displaystyle \sum_{i=1}^n{w}_{ik}^2+{\sigma}_{dk}^{-2}{\sigma}^2}\right)}^{-1}{\displaystyle \sum_{i=1}^n{w}_{ik}}\left({y}_i-\mu -{\displaystyle \sum_{k=1}^p{x}_{ik}{a}_k-{\displaystyle \sum_{k^{\prime}\ne k}^p{w}_{ik\prime }{d}_{k\prime }-{\displaystyle \sum_{k=1}^t{\varOmega}_{ik}{\varTheta}_k}}}\right),{\left({\displaystyle \sum_{i=1}^n{w}_{ik}^2+{\sigma}_{dk}^{-2}{\sigma}^2}\right)}^{-1}{\sigma}^2\right] $$

The conditional posterior related to additive-additive epistasis is given by

11 $$ p\left(a{a}_k\Big|\dots \right)\propto {e}^{-0.5{\sigma}^{-2}{\left[a{a}_k-{\left({\displaystyle \sum_{i=1}^n{\eta}_{ik}^2+{\sigma}_{aak}^{-2}{\sigma}^2}\right)}^{-1}{\displaystyle \sum_{i=1}^n{\eta}_{ik}}\left({\overset{\smile }{y}}_i-{\displaystyle \sum_{k=1:\kern0.5em a{a}_k\neg \ni \left\{\varOmega, \varTheta \right\}}^t{\varOmega}_{ik}{\varTheta}_k}\right)\right]}^T\left({\displaystyle \sum_{i=1}^n{\eta}_{ik}^2+{\sigma}_{aak}^{-2}{\sigma}^2}\right)\left[a{a}_k-{\left({\displaystyle \sum_{i=1}^n{\eta}_{ik}^2+{\sigma}_{aak}^{-2}{\sigma}^2}\right)}^{-1}{\displaystyle \sum_{i=1}^n{\eta}_{ik}}\left({\overset{\smile }{y}}_i-{\displaystyle \sum_{k=1:\kern0.5em a{a}_k\neg \ni \left\{\varOmega, \varTheta \right\}}^t{\varOmega}_{ik}{\varTheta}_k}\right)\right]} $$

where $\overset{\smile }{y}={y}_i-\mu -{\displaystyle \sum_{k=1}^p{x}_{ik}{a}_k-{\displaystyle \sum_{k=1}^p{w}_{ik}{d}_k}}$ and ¬ ∍ {*Ω*, *Θ*} mean *aa*_*k*_ is not contained as a member of the *Ω*_*k*_ matrix and *Θ*_*k*_ vector. The additive-additive epistasis is also normally distributed, presenting a mean equal to ${\left({\displaystyle \sum_{i=1}^n{\eta}_{ik}^2+{\sigma}_{aak}^{-2}{\sigma}^2}\right)}^{-1}{\displaystyle \sum_{i=1}^n{\eta}_{ik}}\left({\overset{\smile }{y}}_i-{\displaystyle \sum_{k=1:\kern0.5em a{a}_k\neg \ni \left\{\varOmega, \varTheta \right\}}^t{\varOmega}_{ik}{\varTheta}_k}\right)$ and variance equal to ${\left({\displaystyle \sum_{i=1}^n{\eta}_{ik}^2+{\sigma}_{aak}^{-2}{\sigma}^2}\right)}^{-1}{\sigma}^{-2}$. The conditional posterior distributions for the others epistatic effects are also Gaussian distribution, as given by

12 $$ p\left(a{d}_k\Big|\dots \right)\propto {e}^{-0.5{\sigma}^{-2}{\left[a{d}_k-{\left({\displaystyle \sum_{i=1}^n{\gamma}_{ik}^2+{\sigma}_{adk}^{-2}{\sigma}^2}\right)}^{-1}{\displaystyle \sum_{i=1}^n{\gamma}_{ik}}\left({\overset{\smile }{y}}_i-{\displaystyle \sum_{k=1:\kern0.5em a{d}_k\neg \ni \left\{\varOmega, \varTheta \right\}}^t{\varOmega}_{ik}{\varTheta}_k}\right)\right]}^T\left({\displaystyle \sum_{i=1}^n{\gamma}_{ik}^2+{\sigma}_{adk}^{-2}{\sigma}^2}\right)\left[a{d}_k-{\left({\displaystyle \sum_{i=1}^n{\gamma}_{ik}^2+{\sigma}_{adk}^{-2}{\sigma}^2}\right)}^{-1}{\displaystyle \sum_{i=1}^n{\gamma}_{ik}}\left({\overset{\smile }{y}}_i-{\displaystyle \sum_{k=1:\kern0.5em a{d}_k\neg \ni \left\{\varOmega, \varTheta \right\}}^t{\varOmega}_{ik}{\varTheta}_k}\right)\right]} $$

13 $$ p\left(d{a}_k\Big|\dots \right)={e}^{-0.5{\sigma}^{-2}{\left[d{a}_k-{\left({\displaystyle \sum_{i=1}^n{\varpi}_{ik}^2+{\sigma}_{dak}^{-2}{\sigma}^2}\right)}^{-1}{\displaystyle \sum_{i=1}^n{\varpi}_{ik}}\left({\overset{\smile }{y}}_i-{\displaystyle \sum_{k=1:\kern0.5em d{a}_k\neg \ni \left\{\varOmega, \varTheta \right\}}^t{\varOmega}_{ik}{\varTheta}_k}\right)\right]}^T\left({\displaystyle \sum_{i=1}^n{\varpi}_{ik}^2+{\sigma}_{dak}^{-2}{\sigma}^2}\right)\left[d{a}_k-{\left({\displaystyle \sum_{i=1}^n{\varpi}_{ik}^2+{\sigma}_{dak}^{-2}{\sigma}^2}\right)}^{-1}{\displaystyle \sum_{i=1}^n{\varpi}_{ik}}\left({\overset{\smile }{y}}_i-{\displaystyle \sum_{k=1:\kern0.5em d{a}_k\neg \ni \left\{\varOmega, \varTheta \right\}}^t{\varOmega}_{ik}{\varTheta}_k}\right)\right]} $$

14 $$ p\left(d{d}_k\Big|\dots \right)\propto {e}^{-0.5{\sigma}^{-2}{\left[d{d}_k-{\left({\displaystyle \sum_{i=1}^n{\varphi}_{ik}^2+{\sigma}_{ddk}^{-2}{\sigma}^2}\right)}^{-1}{\displaystyle \sum_{i=1}^n{\varphi}^{ik}}\left({\overset{\smile }{y}}_i-{\displaystyle \sum_{k=1:\kern0.5em d{d}_k\neg \ni \left\{\varOmega, \varTheta \right\}}^t{\varOmega}_{ik}{\varTheta}_k}\right)\right]}^T\left({\displaystyle \sum_{i=1}^n{\varphi}_{ik}^2+{\sigma}_{ddk}^{-2}{\sigma}^2}\right)\left[d{d}_k-{\left({\displaystyle \sum_{i=1}^n{\varphi}_{ik}^2+{\sigma}_{ddk}^{-2}{\sigma}^2}\right)}^{-1}{\displaystyle \sum_{i=1}^n{\varphi}_{ik}}\left({\overset{\smile }{y}}_i-{\displaystyle \sum_{k=1:\kern0.5em d{d}_k\neg \ni \left\{\varOmega, \varTheta \right\}}^t{\varOmega}_{ik}{\varTheta}_k}\right)\right]} $$

The conditional distributions related to the variance components related to additive, dominant and epistatic effects are given by

15 $$ p\left({\sigma}_{a_k}^2\Big|\dots \right)=p\left({\sigma}_{a_k}^2\right)p\left({a}_k\Big|{\sigma}_{a_k}^2\right)\propto {\left({\sigma}_{a_k}^2\right)}^{-\left(\frac{n_a}{2}-1\right)}{e}^{-\frac{{a_k}^2}{2{\sigma}_{a_k}^2}} $$

16 $$ p\left({\sigma}_{d_k}^2\Big|\dots \right)=p\left({\sigma}_{d_k}^2\right)p\left({d}_k\Big|{\sigma}_{d_k}^2\right)\propto {\left({\sigma}_{d_k}^2\right)}^{-\left(\frac{n_a}{2}-1\right)}{e}^{-\frac{{d_k}^2}{2{\sigma}_{d_k}^2}} $$

17 $$ p\left({\sigma}_{a{a}_k}^2\Big|\dots \right)=p\left({\sigma}_{a{a}_k}^2\right)p\left(a{a}_k\Big|{\sigma}_{a{a}_k}^2\right)\propto {\left({\sigma}_{a{a}_k}^2\right)}^{-\left(\frac{n_a}{2}-1\right)}{e}^{-\frac{a{a_k}^2}{2{\sigma}_{a{a}_k}^2}} $$

18 $$ p\left({\sigma}_{a{d}_k}^2\Big|\dots \right)=p\left({\sigma}_{a{d}_k}^2\right)p\left(a{d}_k\Big|{\sigma}_{a{d}_k}^2\right)\propto {\left({\sigma}_{a{d}_k}^2\right)}^{-\left(\frac{n_a}{2}-1\right)}{e}^{-\frac{a{d_k}^2}{2{\sigma}_{a{d}_k}^2}} $$

19 $$ p\left({\sigma}_{d{a}_k}^2\Big|\dots \right)=p\left({\sigma}_{d{a}_k}^2\right)p\left(d{a}_k\Big|{\sigma}_{d{a}_k}^2\right)\propto {\left({\sigma}_{d{a}_k}^2\right)}^{-\left(\frac{n_a}{2}-1\right)}{e}^{-\frac{d{a_k}^2}{2{\sigma}_{d{a}_k}^2}} $$

20 $$ p\left({\sigma}_{d{d}_k}^2\Big|\dots \right)=p\left({\sigma}_{d{d}_k}^2\right)p\left(d{d}_k\Big|{\sigma}_{d{d}_k}^2\right)\propto {\left({\sigma}_{d{d}_k}^2\right)}^{-\left(\frac{n_a}{2}-1\right)}{e}^{-\frac{d{d_k}^2}{2{\sigma}_{d{d}_k}^2}} $$

where *n*_*a*_ is the corrected degree of freedom related to the Xu [37] model. These distributions can be recognized as inverse-scaled chi-square distributions with *n*_*a*_ degrees of freedom and scale equal to the square of the QTL effect, that is, *a*_*k*_^2^, *d*_*k*_^2^, *aa*_*k*_^2^, *ad*_*k*_^2^, *da*_*k*_^2^, *dd*_*k*_^2^ for principal and epistatic effects, respectively.

Given the kk` pair of QTL, the epistatic combination could be sampled (without replacement) from a uniform distribution ranging from 0 to *p* (number of marker intervals). After sampling the pair of QTL responsible for the candidate epistasis, the variables *η*_*ik´*_, *γ*_*ik´*_, *ϖ*_*ik´*_ and *φ*_*ik´*_ are obtained by the element-wise product of the additive and dominant matrices considering the respective QTL, i.e., it was built by the dot product given by (*x*_*ik*_, *w*_*ik*_) ∘ (*x*_*ik´*_, *w*_*ik´*_), where *k* ≠ *k* `.

In RJMCMC for QTL analysis, the change of variable is unnecessary because the determinant is equal to one [41, 43–45]. Thus, the decision rule to add a new epistatic interaction in the model is given by the decision rule min[1, *α*(*t*, *t* + 1)]_,_ where

21 $$ \alpha \left(t,t+1\right)=\frac{{\displaystyle \prod_{i=1}^np\left(y\Big|t+1\right)p\left(t+1\Big|\phi \right)}\xi \left(t,t+1\right)}{{\displaystyle \prod_{i=1}^np\left(y\Big|t\right)p\left(t\Big|\phi \right)}\xi \left(t+1,t\right)} $$

Here, *t* is the current number of epistases in the model, *t* + 1 is the candidate model with the new epistatic interaction added, and *p*(*t*|*ϕ*) is the prior truncated Poisson distribution given previously by eq (6). The Hastings-proposed distribution *ξ*(*t*, *t* + 1) is necessary to allow the reversible process in the model during the MCMC process. It is given by *ξ*(*t*, *t* + 1) = *p*_*a*_ and $\xi \left(t+1,t\right)=\frac{1}{t+1}{p}_d$, where the prior probability to add, delete or keep the number of epistatic terms in the model is *p*_*a*_ = *p*_*d*_ = *p*_0_ = 1/3, respectively [39]. The term $\frac{1}{t+1}$ is the probability that the kth candidate epistasis would be deleted after being included in the model (the reverse probability). If the candidate epistasis is included in the model, the conditional distribution of the data (likelihood) described in the numerator of the decision rule that includes the new epistasis *p*(*y*|*t* + 1) is given by

22 $$ p\left(y\Big|t+1\right)\propto {e}^{-0.5{\sigma}^{-2}{\left({y}_i-\mu -{\displaystyle \sum_{k=1}^p{x}_{ik\prime }{a}_{k\prime }-{\displaystyle \sum_{k=1}^p{w}_{ik}{d}_k}-{\displaystyle \sum_{k=0}^t{\Omega}_{ik}{\Theta}_k}}+{\omega}_{t+1}{\rho}_{t+1}\right)}^T\left({y}_i-\mu -{\displaystyle \sum_{k=1}^p{x}_{ik\prime }{a}_{k\prime }-{\displaystyle \sum_{k=1}^p{w}_{ik}{d}_k}-{\displaystyle \sum_{k=0}^t{\varOmega}_{ik}{\varTheta}_k}}+{\omega}_{t+1}{\rho}_{t+1}\right)} $$

where $$ {\omega}_{t+1}{\rho}_{t+1}=\left(\begin{array}{cccc}\hfill {\eta}_{t+1}\hfill & \hfill {\gamma}_{t+1}\hfill & \hfill {\varpi}_{t+1}\hfill & \hfill {\varphi}_{t+1}\hfill \end{array}\right){\left(\begin{array}{cccc}\hfill a{a}_{t+1}\hfill & \hfill a{d}_{t+1}\hfill & \hfill d{a}_{t+1}\hfill & \hfill d{d}_{t+1}\hfill \end{array}\right)}^T $$. The likelihood given the unchanged dimension *p*(*y*|*t*) is provided by

23 $$ p\left(y\Big|t\right)\propto {e}^{-0.5{\sigma}^{-2}{\left({y}_i-\mu -{\displaystyle \sum_{k=1}^p{x}_{ik\prime }{a}_{k\prime }-{\displaystyle \sum_{k=1}^p{w}_{ik}{d}_k}-{\displaystyle \sum_{k=0}^t{\varOmega}_{ik}{\varTheta}_k}}\right)}^T\left({y}_i-\mu -{\displaystyle \sum_{k=1}^p{x}_{ik\prime }{a}_{k\prime }-{\displaystyle \sum_{k=1}^p{w}_{ik}{d}_k}-{\displaystyle \sum_{k=0}^t{\varOmega}_{ik}{\varTheta}_k}}\right)} $$

Thus, the Bayes factor criterion [*α*(*t*, *t* + 1)] to accept the new epistasis is given by

24 $$ \alpha \left(t,t+1\right)=\frac{{\displaystyle \prod_{i=1}^np\left(y\Big|t+1\right)}}{{\displaystyle \prod_{i=1}^np\left(y\Big|t\right)}}\frac{\phi }{t+1}\frac{p_d}{\left(t+1\right){p}_a} $$

where *ϕ* is the Poisson mean in $p\left(t\Big|\phi \right)\propto \frac{\phi^t{e}^{-\phi }}{t!}$.

Therefore, if *α*(*t*, *t* + 1) is greater than a random variable sampled from a uniform distribution [0, 1], then the new epistasis is accepted in the model; otherwise, the epistasis number is maintained.

After deciding whether to insert a new QTL, we could decide if the current epistasis dimension of the model could decrease from *t* to *t* − 1. For this, we sample a candidate epistasis in the model ranging from 1 to *t,* and the *k*th epistasis is deleted from the model using the following decision rule:

25 $$ \alpha \left(t,t-1\right)=\frac{{\displaystyle \prod_{i=1}^np\left(y\Big|t\right)p\left(t\Big|\phi \right)\xi}\left(t,t-1\right)}{{\displaystyle \prod_{i=1}^np\left(y\Big|t-1\right)p\left(t-1\Big|\phi \right)}\xi \left(t-1,t\right)} $$

where *p*(*y*|*t*) has been described previously.

Here, *p*(*y*|*t* − 1) is a data conditional distribution given the deleted epistasis. It can be described by

26 $$ p\left(y\Big|t-1\right)\propto {e}^{-0.5{\sigma}^{-2}{\left({y}_i-\mu -{\displaystyle \sum_{k=1}^p{x}_{ik\prime }{a}_{k\prime }-{\displaystyle \sum_{k=1}^p{w}_{ik}{d}_k}-{\displaystyle \sum_{k=0}^{t-1}{\varOmega}_{ik}{\varTheta}_k}}\right)}^T\left({y}_i-\mu -{\displaystyle \sum_{k=1}^p{x}_{ik\prime }{a}_{k\prime }-{\displaystyle \sum_{k=1}^p{w}_{ik}{d}_k}-{\displaystyle \sum_{k=0}^{t-1}{\varOmega}_{ik}{\varTheta}_k}}\right)} $$

Therefore, the Bayes factor for the decision rule is provided by

27 $$ \alpha \left(t,t-1\right)=\frac{{\displaystyle \prod_{i=1}^np\left(y\Big|t\right)}}{{\displaystyle \prod_{i=1}^np\left(y\Big|t-1\right)}}\frac{t}{\phi}\frac{p_at}{p_d} $$

Therefore, if *α*(*t*, *t* − 1) is greater than a random variable sampled from a uniform distribution [0, 1], then the current sampled epistasis is deleted from the model; otherwise, the number of epistases is maintained. Here, given any epistatic QTL pair in *t,* the proposed probability of deleting a specific pair is $\frac{1}{t}\times {p}_d$, and the reversible term is given by *p*_*a*_.

The next step is to sample the residual variance from

28 $$ p\left({\sigma}^2\Big|\dots \right)=p\left({\sigma}^2\right)p\left(y\Big|b,a,d,\varTheta, x,w,{\sigma}^2\right)\propto {\left({\sigma}^2\right)}^{-\left(\frac{n_a-1+n}{2}-1\right)}{e}^{-\frac{e\prime e}{2{\sigma}^2}} $$

This probability is also an inverse-scaled chi-square distribution with *n*_*a*_ + *n* − 1 degrees of freedom and scale equal to the residual sum square *e´e*, where $e={y}_i-\mu -{\displaystyle \sum_{k=1}^p{x}_{ik\prime }{a}_{k\prime }-{\displaystyle \sum_{k=1}^p{w}_{ik}{d}_k}-{\displaystyle \sum_{k=0}^t{\varOmega}_{ik}{\varTheta}_k}}$

Given the new parameters sampled from their conditional distribution, the next step is to sample the QTL genotype from it posterior given by

29 $$ p\left({x}_{ik}\Big|\dots \right)=\frac{p\left({x}_{ik}=g\right){H}_{kL}\left(g,{m}_l\right){H}_{kR}\left(g,{m}_R\right)p\left(y\Big|b,a,d,\varTheta, x,w,{\sigma}^2\right)}{{\displaystyle \sum_{z=1}^3p\left({x}_{ik}={g}_z\right){H}_{kL}\left({g}_z,{m}_l\right){H}_{kR}\left({g}_z,{m}_R\right)p\left(y\Big|b,a,d,\varTheta, x,w,{\sigma}^2\right)}} $$

where *p*(*x*_*ik*_ = *g*) is the prior probability of the segregating QTL genotype in a structured population. For example, in F_2:3,_ as used in the presented study, *p*(*x*_*ik*_ = 1) = *p*(*x*_*ik*_ = − 1) = 3/8 and *p*(*x*_*ik*_ = 0) = 1/4 or 1/4, 1/4 and 1/2 for F_2_ populations, respectively. *H*_*kL*_(*g*, *m*_*l*_) and *H*_*kR*_(*g*, *m*_*R*_) are Markov transitional matrices between the markers *M*_*k*_^*L*^ and *M*_*k*_^*R*^ and the QTL Q_k_. These matrices are built based on the conditional posterior of QTL given the marker genotype. For example, in the F_2:3_ data given the marker coded as 1 (MM), the conditional probability of obtaining the QTL coded as 1 (QQ) in F_2:3_ is (1 − *r*)^2^ + 0.5*r*(1 − *r*).

The position of the QTL in the genome does not present a closed-form; consequently, the Metropolis–Hastings (M-H) algorithm is requested. For this, we used a uniform distribution as the proposed distribution to sample *λ*.

The M-H algorithm does not require that the parameter has a known probability function and, instead, makes use of an auxiliary function that can be sampled and where the candidate values can be accepted with α probability. In the case of interval mapping, a uniform distribution can be used as an auxiliary function that is sampled across each interval delimited by *max*(*λ*_*j* − 1_, *λ*_*j*_ + *d*) and *min*(*λ*_*j* + 1_, *λ*_*j*_ − *d*), where *d* is a constant that defines the tuning within the interval j, usually fixed between 1 and 2 cM. The Hastings corrections are denoted by *u*(λ*, *λ*), and the new position will be accepted in the kth iteration with probability *min*(1, *α*), where *α* is given by

30 $$ \alpha =\frac{p\left({\lambda}_j^{*}\Big|y,b,a,d,\varTheta, x,w,{\sigma}^2\right)u\left({\lambda}_j^{*},{\lambda}_j\right)}{p\left({\lambda}_j^0\Big|y,b,a,d,\varTheta, x,w,{\sigma}^2\right)u\left({\lambda}_j,{\lambda}_j^{*}\right)} $$

Finally, the posterior conditional probability distribution for the average number of epistatic effects (*ϕ*) in the model can be given by

31 $$ p\left(\phi \Big|\dots \right)=p\left(\phi \right)p\left(t\Big|\phi \right)\propto {\phi}^t{e}^{-2\phi } $$

This function could be recognized as a gamma distribution presenting a shape parameter equal to *t* + 1 and a rate equal to 2.

### The RJMCMC algorithm

The reversible-jump Monte Carlo Markov Chain (RJMCMC) algorithm is given as follows:

Initiate the RJMCMC algorithm using only the additive-dominant model (*t = 0*) and using the following initial guess (*a*_1_^0^, *a*_2_^0^.... *a*_*p*_^0^, *d*_1_^0^, *d*_2_^0^ … *d*_*p*_^0^, *Θ* ^0^ = 0), $\left({\sigma_{a_1}^2}^0,{\sigma_{a_2}^2}^0,....{\sigma_{a_p}^2}^0,{\sigma_{d_1}^2}^0,{\sigma_{d_2}^2}^0\dots {\sigma_{d_p}^2}^0=\frac{\operatorname{var}(y)*{1}^{-5}}{p}\right)$, $$ {\sigma^2}^{{}^0}=\operatorname{var}(y),{\phi}^0=\left(\begin{array}{c}\hfill p\hfill \\ {}\hfill 2\hfill \end{array}\right)*0.01 $$ and $\operatorname{var}\left({\varTheta}^0\right)=\left\{{\sigma}_{a{a}_k}^2,{\sigma}_{a{d}_k}^2,{\sigma}_{d{a}_k}^2,{\sigma}_{d{d}_k}^2\right\}=\operatorname{var}(y)*{1}^{-5}$. It is worth highlighting that the initial guess for the epistasis *Θ* = 0 and the variance of epistatic effects [var(*Θ*)] are used only when *t* ≥ 1. The variances of the additive, dominant and epistatic effects were initiated with values near to 0 heritability. The initial guess for *ϕ* ^0^ is based on 1% of all pairwise epistasis. For the QTL additive matrix *x*_*ik*_, the initial guess is based on the position *λ* taken as the midpoint of the interval; ignoring the likelihood, that is

$$ p\left({x}_{ik}\Big|\dots \right)=\frac{p\left({x}_{ik}=g\right){H}_{kL}\left(g,{m}_l\right){H}_{kR}\left(g,{m}_R\right)}{{\displaystyle \sum_{z=1}^3p\left({x}_{ik}={g}_z\right){H}_{kL}\left({g}_z,{m}_l\right){H}_{kR}\left({g}_z,{m}_R\right)}} $$

This conditional is slightly different from (30) because all genetic parameters in the likelihood are assumed as 0 in the first MCMC round.

**Fig. 12:**
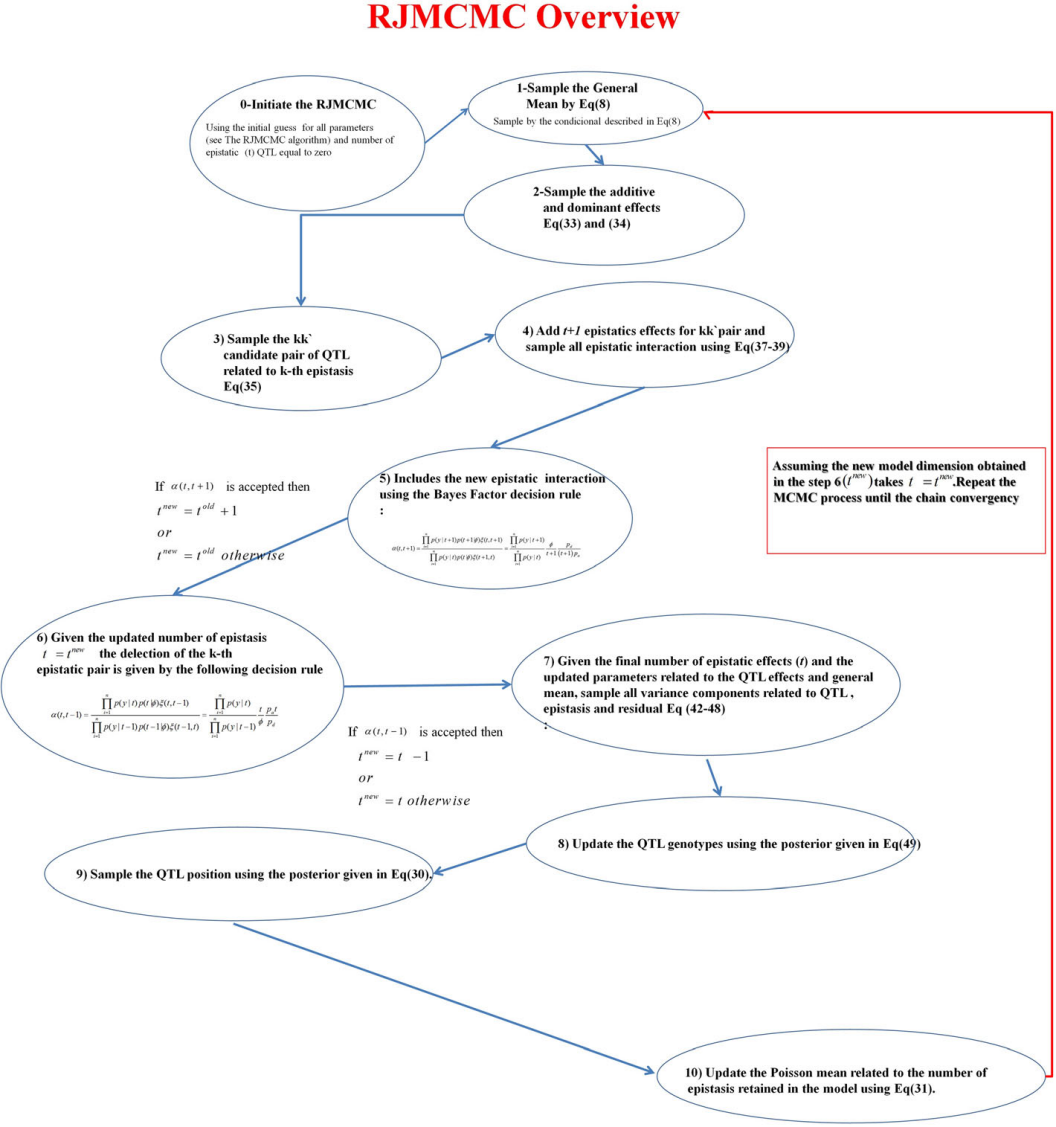
Overview of the RJMCMC algorithm

1. First, we started the RJMCMC using a non-epistatic model (*t = 0*). The global mean was sampled from a normal distribution presenting the following parameters:
  32 $$ p\left(\mu \Big|....\right)\propto N\left[\frac{1}{n}{\displaystyle \sum_{i=1}^{n_j}\left({y}_i-{\displaystyle \sum_{k=1}^p{x}_{ik}{a}_k-{\displaystyle \sum_{k=1}^p{w}_{ik}{d}_k-{\displaystyle \sum_{k=1}^t{\varOmega}_{ik}{\varTheta}_k}}}\right)},\frac{\sigma^2}{n}\right] $$
  where *p*(|....) means conditional on others parameters
2. Using the new value of general mean, we sampled the additive effects from a normal distribution given by
  33 $$ p\left({a}_k\Big|\dots \right)\propto N\left[{\left({\displaystyle \sum_{i=1}^n{x}_{ik}^2+{\sigma}_{ak}^{-2}{\sigma}^2}\right)}^{-1}{\displaystyle \sum_{i=1}^n{x}_{ik}}\left({y}_i-\mu -{\displaystyle \sum_{k^{\prime}\ne k}^p{x}_{ik\prime }{a}_{k\prime }-{\displaystyle \sum_{k=1}^p{w}_{ik}{d}_k}-{\displaystyle \sum_{k=1}^t{\varOmega}_{ik}{\varTheta}_k}}\right),{\left({\displaystyle \sum_{i=1}^n{x}_{ik}^2+{\sigma}_{ak}^{-2}{\sigma}^2}\right)}^{-1}{\sigma}^2\right] $$
3. Next, we sampled the dominant effects from:
  34 $$ p\left({d}_k\Big|\dots \right)\propto N\left[{\left({\displaystyle \sum_{i=1}^n{w}_{ik}^2+{\sigma}_{dk}^{-2}{\sigma}^2}\right)}^{-1}{\displaystyle \sum_{i=1}^n{w}_{ik}}\left({y}_i-\mu -{\displaystyle \sum_{k=1}^p{x}_{ik}{a}_k-{\displaystyle \sum_{k^{\prime}\ne k}^p{w}_{ik\prime }{d}_{k\prime }-{\displaystyle \sum_{k=1}^t{\varOmega}_{ik}{\varTheta}_k}}}\right),{\left({\displaystyle \sum_{i=1}^n{w}_{ik}^2+{\sigma}_{dk}^{-2}{\sigma}^2}\right)}^{-1}{\sigma}^2\right] $$
4. Adding the *t* + 1-th epistatic effect for *k* ≠ *k* ` locus sampling, k and k` from a discrete uniform distribution *ranges from* [1 *to p*]. The use of sampling without replacement is to guarantee an exhaustive search in epistatic space. Therefore, for each pair of QTL, we sampled the following criteria:
  35 $$ kk`=\left\{\begin{array}{c}\hfill \left[ uniform\kern0.5em \left(1\kern0.5em to\kern0.5em p\right),\kern0.5em  uniform\kern0.5em \left(1\kern0.5em to\kern0.5em p`\right)\right]\kern3em  if\kern1.5em t=0\kern0.5em \hfill \\ {}\hfill {I}_{kk`}\Big[ uniform\kern0.5em \left(1\kern0.5em to\kern0.5em p\right),\kern0.5em  uniform\kern0.5em \left(1\kern0.5em to\kern0.5em p`\right)\kern2em  if\kern1.5em t>=1\kern0.5em \hfill \end{array}\right. $$
  where the indicator function *I*_*kk* `_ = 1 if *kk* ` ¬ ∍ {1 … *t*}
5. Given the pair kk´, sampling the *kth* additive-by-additive, additive-by-dominant, dominant-by-additive and dominant-by-dominant, respectively, from
  36 $$ p\left(a{a}_k\Big|\dots \right)\propto N\left[{\left({\displaystyle \sum_{i=1}^n{\eta}_{ik}^2+{\sigma}_{aak}^{-2}{\sigma}^2}\right)}^{-1}{\displaystyle \sum_{i=1}^n{\eta}_{ik}}\left({\overset{\smile }{y}}_i-{\displaystyle \sum_{k=1:\kern0.5em a{a}_k\neg \ni \left\{\varOmega, \varTheta \right\}}^t{\varOmega}_{ik}{\varTheta}_k}\right),{\left({\displaystyle \sum_{i=1}^n{\eta}_{ik}^2+{\sigma}_{aak}^{-2}{\sigma}^2}\right)}^{-1}{\sigma}^2\right] $$
  37 $$ p\left(a{d}_k\Big|\dots \right)=N\left[{\left({\displaystyle \sum_{i=1}^n{\gamma}_{ik}^2+{\sigma}_{adk}^{-2}{\sigma}^2}\right)}^{-1}{\displaystyle \sum_{i=1}^n{\gamma}_{ik}}\left({\overset{\smile }{y}}_i-{\displaystyle \sum_{k=1:\kern0.5em a{d}_k\neg \ni \left\{\varOmega, \varTheta \right\}}^t{\varOmega}_{ik}{\varTheta}_k}\right),{\left({\displaystyle \sum_{i=1}^n{\gamma}_{ik}^2+{\sigma}_{adk}^{-2}{\sigma}^2}\right)}^{-1}{\sigma}^2\right] $$
  38 $$ p\left(d{a}_k\Big|\dots \right)=N\left[{\left({\displaystyle \sum_{i=1}^n{\varpi}_{ik}^2+{\sigma}_{dak}^{-2}{\sigma}^2}\right)}^{-1}{\displaystyle \sum_{i=1}^n{\varpi}_{ik}}\left({\overset{\smile }{y}}_i-{\displaystyle \sum_{k=1:\kern0.5em d{a}_k\neg \ni \left\{\varOmega, \varTheta \right\}}^t{\varOmega}_{ik}{\varTheta}_k}\right),{\left({\displaystyle \sum_{i=1}^n{\varpi}_{ik}^2+{\sigma}_{dak}^{-2}{\sigma}^2}\right)}^{-1}{\sigma}^2\right] $$
  39 $$ p\left(d{d}_k\Big|\dots \right)=N\left[{\left({\displaystyle \sum_{i=1}^n{\varphi}_{ik}^2+{\sigma}_{ddk}^{-2}{\sigma}^2}\right)}^{-1}{\displaystyle \sum_{i=1}^n{\varphi}_{ik}}\left({\overset{\smile }{y}}_i-{\displaystyle \sum_{k=1:\kern0.5em d{d}_k\neg \ni \left\{\varOmega, \varTheta \right\}}^t{\varOmega}_{ik}{\varTheta}_k}\right),{\left({\displaystyle \sum_{i=1}^n{\varphi}_{ik}^2+{\sigma}_{ddk}^{-2}{\sigma}^2}\right)}^{-1}{\sigma}^2\right] $$
  where $\overset{\smile }{y}={y}_i-\mu -{\displaystyle \sum_{k=1}^p{x}_{ik}{a}_k-{\displaystyle \sum_{k=1}^p{w}_{ik}{d}_k}}$ and ¬ ∍ {*Ω*, *Θ*} means it is not contained as a member of the *Ω*_*k*_ matrix and the *Θ*_*k*_ vector.
6. Acceptance of the new epistatic effect, with probability *ρ*, is given by min[1, *α*(*t*, *t* + 1)], where
  40 $$ \alpha \left(t,t+1\right)=\frac{{\displaystyle \prod_{i=1}^np\left(y\Big|t+1\right)p\left(t+1\Big|\phi \right)}\xi \left(t,t+1\right)}{{\displaystyle \prod_{i=1}^np\left(y\Big|t\right)p\left(t\Big|\phi \right)}\xi \left(t+1,t\right)}=\frac{{\displaystyle \prod_{i=1}^np\left(y\Big|t+1\right)}}{{\displaystyle \prod_{i=1}^np\left(y\Big|t\right)}}\frac{\phi }{t+1}\frac{p_d}{\left(t+1\right){p}_a} $$
  is the Bayes factor (BF) and *ξ*(*t*, *t* + 1) = *p*_*a*_ and $\xi \left(t+1,t\right)=\frac{1}{t+1}{p}_d$ are proposed probabilities based on the Hastings’ adjustment. The conditional distributions presented in the numerator *p*(*y*|*t* + 1), denominator *p*(*y*|*t*) *and p*(*t*|*ϕ*) of the BF decision rule are described in (22), (23) and (6), respectively. If the kth epistasis is accepted, then *t* ^*new*^ = *t* ^*old*^ + 1; otherwise, *t* ^*new*^ = *t* ^*old*^.
7. In the next step, if t > 0, any epistasis present in the updated model (*t* = *t* ^*new*^) can be deleted, and the change in model dimension from *t* to *t - 1* is given as follows. Given *t*, the candidate epistasis to be deleted is labeled from 1 to *t* and sampled from a discrete uniform distribution ranging from [1 *to t*]. Here, the proposed probability is slightly different from adding the epistatic effects because the updated epistatic space is now output *t* from step 5. Thus, the probability of deleting the k^th^ epistatic effect is given by the decision rule min[1, *α*(*t*, *t* − 1)]_,_ where
  41 $$ \alpha \left(t,t-1\right)=\frac{{\displaystyle \prod_{i=1}^np\left(y\Big|t\right)p\left(t\Big|\phi \right)}\xi \left(t,t-1\right)}{{\displaystyle \prod_{i=1}^np\left(y\Big|t-1\right)p\left(t-1\Big|\phi \right)}\xi \left(t-1,t\right)}=\frac{{\displaystyle \prod_{i=1}^np\left(y\Big|t\right)}}{{\displaystyle \prod_{i=1}^np\left(y\Big|t-1\right)}}\frac{t}{\phi}\frac{p_at}{p_d} $$
  and *ξ*(*t*, *t* − 1) = *p*_*a*_, $\xi \left(t-1,t\right)={p}_d\times \frac{1}{t}$ and *p*(*y*|*t* − 1) are described in (26). If *α*(*t*, *t* − 1) is accepted, then *t* ^*new*^ = *t* − 1; otherwise, *t* ^*new*^ = *t*_,_ where *t* is the output from step 5.
8. If any epistatic term is kept in the model, its variance plus additive, dominance and residual variances may be sampled from inverted-scale, chi-squared distribution as follows:
  42 $$ p\left({\sigma}_{a_k}^2\Big|\dots \right)\sim Inv- scaled-{\chi}^2\left({n}_a,{a}_k^2\right) $$
  43 $$ p\left({\sigma}_{d_k}^2\Big|\dots \right)\sim Inv- scaled-{\chi}^2\left({n}_a,{d}_k^2\right) $$
  44 $$ p\left({\sigma}_{a{a}_k}^2\Big|\dots \right)\sim Inv- scaled-{\chi}^2\left({n}_a,a{a}_k^2\right) $$
  45 $$ p\left({\sigma}_{a{d}_k}^2\Big|\dots \right)\sim Inv- scaled-{\chi}^2\left({n}_a,a{d}_k^2\right) $$
  46 $$ p\left({\sigma}_{d{a}_k}^2\Big|\dots \right)\sim Inv- scaled-{\chi}^2\left({n}_a,d{a}_k^2\right) $$
  47 $$ p\left({\sigma}_{d{d}_k}^2\Big|\dots \right)\sim Inv- scaled-{\chi}^2\left({n}_a,d{d}_k^2\right) $$
  48 $$ p\left({\sigma}^2\Big|\dots \right)\sim Inv- scaled-{\chi}^2\left({n}_a+n-1,e\acute{\mkern6mu}e\right) $$
  where *e´e* is the residual sum square and *n*_*a*_ is the corrected degree of freedom related to prior variance distribution.
9. Given the updated values of parameters, the QTL genotypes (1, 0 and −1) can be updated given the neighboring markers using the posterior probability
  49 $$ p\left({x}_{ik}\Big|\dots \right)=\frac{p\left({x}_{ik}=g\right){H}_{kL}\left(g,{m}_l\right){H}_{kR}\left(g,{m}_R\right)p\left({y}_i{}_i\Big|b,a,d,\varTheta, x,w,{\sigma}^2\right)}{{\displaystyle \sum_{z=1}^3p\left({x}_{ik}={g}_z\right){H}_{kL}\left({g}_z,{m}_l\right){H}_{kR}\left({g}_z,{m}_R\right)p\left({y}_i{}_i\Big|b,a,d,\varTheta, x,w,{\sigma}^2\right)}} $$
  where *p*(*x*_*ik*_ = *g*) is the prior probability of the segregating QTL genotype. *H*_*kL*_(*g*, *m*_*l*_) and *H*_*kR*_(*g*, *m*_*R*_) are Markov transitional matrices between the markers *M*_*k*_^*L*^ and *M*_*k*_^*R*^ and the QTL Q_k_. These matrices are built based on the conditional posterior of QTL given the marker genotype.
10. The new QTL position is sampled using the posterior given in (30). *λ* does not present a known position in the genome. Therefore, we can use the Metropolis–Hastings algorithm [11, 24] instead of the Gibbs sampler by using a uniform proposed distribution because *λ* does not present a known distribution.
11. Finally, the Poisson mean *ϕ* is sampled relative to the number of epistatic effects in the model from (31). Because the average number of epistatic terms is critical in the reversible-jump process and unknown, it is given as a random variable. Thus, the new average number of epistatic terms is sampled from a conjugated Gamma–Poisson distribution, i.e., *p*(*ϕ*| …) ∝ *gamma*(*κ* = 1 + *t*, *ς* = 2).
  All RJMCMC processes are summarized in Fig. 12.

#### Post MCMC sampling

##### Principal QTL Effects

The QTL profile was depicted according to the conditional posterior related to the position *f*(*λ*). The length of each linkage group was separated into bins of 3 cM and, for each bin *f*(*λ*), was weighted by the LOD of the maximum posterior within each bin. The LOD posterior was calculated by *LOD* = *W*(*λ*_*i*_)/2 ln(10), where *W*(*λ*_*i*_) is the Wald’s statistic for the *λ*_*i*_ position, i.e., *W*(*λ*) = *a* ^*T*^ *V*_*a*_^− 1^ *a* + *d* ^*T*^ *V*_*d*_^− 1^ *d*.

##### Epistatic QTL Effects

The LODs for epistatic effects were similar to those for additive-dominant effects. Here, all epistatic terms, additive-additive, additive-dominant, dominant-additive and dominant-dominant effects, were used to compose the epistatic LOD. In addition to the LOD, the length of the chain for each epistatic QTL was used as a parameter for selection. The length of the “chain” is an undirected criterion to assess the “strength” of a QTL interaction. The more constant the QTL interaction is in the model throughout the MCMC process, the higher the Bayes factor is, and the more evidence there is for this epistasis relative to the null effect. For this, we assume as reference the final chain length (N) related to the main effects and weighted each LOD based on *LOD* * = *n* × *LOD*/*N*_,_ where *n* is the chain size for the i^th^ epistatic effect. In addition, only epistatic QTL presenting chains larger than 400 were used for LOD analysis. This number was the minimum using the Raftery and Lewis criterion after 10,000 burning and jumping iterations for each 20 samples. Thus, the final chain length was 18,000.

### Simulated data

The simulated dataset was based on the F_2_ population composed of 300 individuals. A total of 104 bi-allelic markers were simulated in seven linkage groups. Six QTL presenting main effects were randomly simulated across the linkage groups. Additionally, seven different epistatic QTL were simulated, totaling 13 QTL controlling the trait. In this scenario, there are 194 additive and dominant candidate effects to be estimated plus 18,624 candidate epistases considering all combinations (additive-additive, additive-dominant, dominant-additive and dominant-dominant). The phenotype was simulated using a heritability of 0.5 and positive effect equal to 2 for additive and dominant effects. The epistatic effects were simulated considering values sampled from a uniform distribution ranging from −2 to 2. The epistasis values were inserted in the Qgene program [15], where we performed all simulations related to the markers and principal QTL effects. It is important to note that the default in the Qgene program for additive and dominant effects are assumed to be equal to 10, but for epistatic QTL, these principal effects are taken as 0. On the other hand, the epistatic effect default is constant and equal to 10; therefore, we prefer to sample the epistasis from an auxiliary uniform distribution. The values related to simulated QTL are given in Additional file 7: Table S3. The residual values were sampled from a Gaussian distribution with a mean of 0 and variance of *σ* ^2^. We compare our method to the Xu and Jia [40] technique using the program PROC QTL [12], which is available in http://statgen.ucr.edu/software.html. The SAS code used in PROC QTL plus our R code and genomic data are available in the Supporting Information (Additional file 8: Texts S1, Additional file 9: Texts S2 and Additional file 10: genomic dataset).

### Real dataset

#### Genetic background

We used 256 F_2:3_ progenies of a maize population derived from the cross of two tropical inbreed lines (L20-01 F and L02-03D). This population, as well as the inbred lines, were developed in the Maize Breeding Program at the Department of Genetics of the Agriculture College “Luiz de Queiroz”. Three F_1_ plants from the cross of these inbred lines were self-crossed to develop the F2 population, which was further self-crossed to develop the F2:3 progenies. The F_2:3_ progenies were evaluated for grain yield in the crop years 2002/2003 and 2003/2004 at the experimental stations of Areão, Caterpillar, Departamento de Genética, ESALQ/USP and Anhembi, all locations scattered across the municipality of Piracicaba-SP in Brazil. The combination of crop year and location was considered as an environment. The experimental design used was a 16x16 lattice with two replicates per environment. Each plot was 4 m in length and 0.8 m between rows. Plots were overplanted and thinned to 20 plants per plot (62,500 plants ha^−1^). The grain yield mean of each plot was adjusted to 15.0% of moisture and corrected for the average stand. In the first stage, the raw data were submitted to joint analysis considering random blocks within replication within environments, genotypes, genotype-by-environment interactions, fixed effects of environments, and replication within environments. In the second stage, the corrected phenotypic means were used for QTL mapping using 139 markers in 10 linkage groups.

To map the maize progenies, we used 139 microsatellite markers. These markers were used to build the linkage group using the software *Mapmaker* 3.0 [18]. The critical LOD score adopted was equal to 3, and the maximal distance between markers was equal to 50 cM. The frequency of recombination was converted to cM using the Kosambi function. The 139 microsatellites markers were distributed through 10 linkage groups that correspond to 10 chromosomes presented in maize species. The total size of the linkage map was 1,858.61 cM, presenting an average distance between markers of 14.41 cM and chromosome length ranging from 103.62 to 303.36 cM for chromosomes 1 and 10, respectively. A simple cross-validation (CV) approach was applied in the real dataset to evaluate the constancy of QTL and its interaction in the model. For this, a 5-fold analysis was implemented in the 256 genotypes forming four groups of 50 entries and one group of 56.

## Additional files

Additional file 1: Figure S1. Genomic profile for QTL in seven simulated linkage groups using RJMCMC for 400 markers. The red dots represent the non-epistatic QTL, and the blue dots represent the simulated epistatic QTL. The dotted line represents the LOD criterion (LOD=3). (JPEG 425 kb)
Additional file 2: Figure S2. Heat map for 79,800 epistatic QTL and chain size in the reversible jumping process. The more red that the epistatic intensity is, the more time the QTL was retained in the MCMC process, meaning its effect on the epistatic complex is more likely. (JPEG 570 kb)
Additional file 3: Figure S3. LOD plot of 79,800 raw epistatic combinations. (JPEG 864 kb)
Additional file 4: Figure S4. 3D plot resolution for the 79,800 weighted epistatic combinations obtained by RJMCMC. The lower triangular plot represents the simulated epistatic effect, and the upper plot represents the estimated epistatic effect. (JPEG 483 kb)
Additional file 5: Table S1. Selected epistases presenting with LOD scores greater than or equal to 3.00 and with chain sizes larger than 500 obtained in the simulated data from 400 markers. Sixteen QTL were simulated, and the first eight were combined pairwise, totaling 28 epistatic interactions. The epistatic QTL were named 58, 57, 80, 140, 208, 194, 279 and 389. (DOCX 13 kb)
Additional file 6: Table S2. Estimated epistases presenting with LOD scores greater than or equal to 3.00, but with chain sizes lower than 500 obtained in the simulated data from 400 markers. This combination was selected because its average distance from the marker combination was lower than 0.1 cM. Sixteen QTL were simulated, and the first eight were combined pairwise, totaling 28 epistatic interaction The epistatic QTL were named 58, 57, 80, 140, 208, 194, 279 and 389. (DOCX 15 kb)
Additional file 7: Table S3 Simulated QTL effects from the Qgene program. (DOCX 14 kb)
Additional file 8:
**Text S1.** R-code. (R 23 kb)
Additional file 9:
**Text S2.** SAS code. (PROC QTL) (SAS 64 kb)
Additional file 10: genomic dataset. (ZIP 9 kb)

## Abbreviations

CV: Cross-validation
DHGLM: Double hierarchical generalized linear models
FDR: False discovery rate
G-BLUP: Genomic Best Linear Unbiased Prediction
LOD: Logarithm of odds
MCMC: Monte Carlo Markov Chain
MDR: Multifactor dimensionality reduction
ML: Machine Learning
QTL: Quantitative Trait Loci
RJMCMC: Reversible Jump, Markov Chain Monte Carlo
vQTL: Variance Quantitative trait loci
