# Supplementary figures and images for: Bayesian reversible-jump for epistasis analysis in genomic studies
Source: BMC Genomics. 2016 Dec 9;17:1012. doi: 10.1186/s12864-016-3342-6 (PMC5148921; doi:10.1186/s12864-016-3342-6)

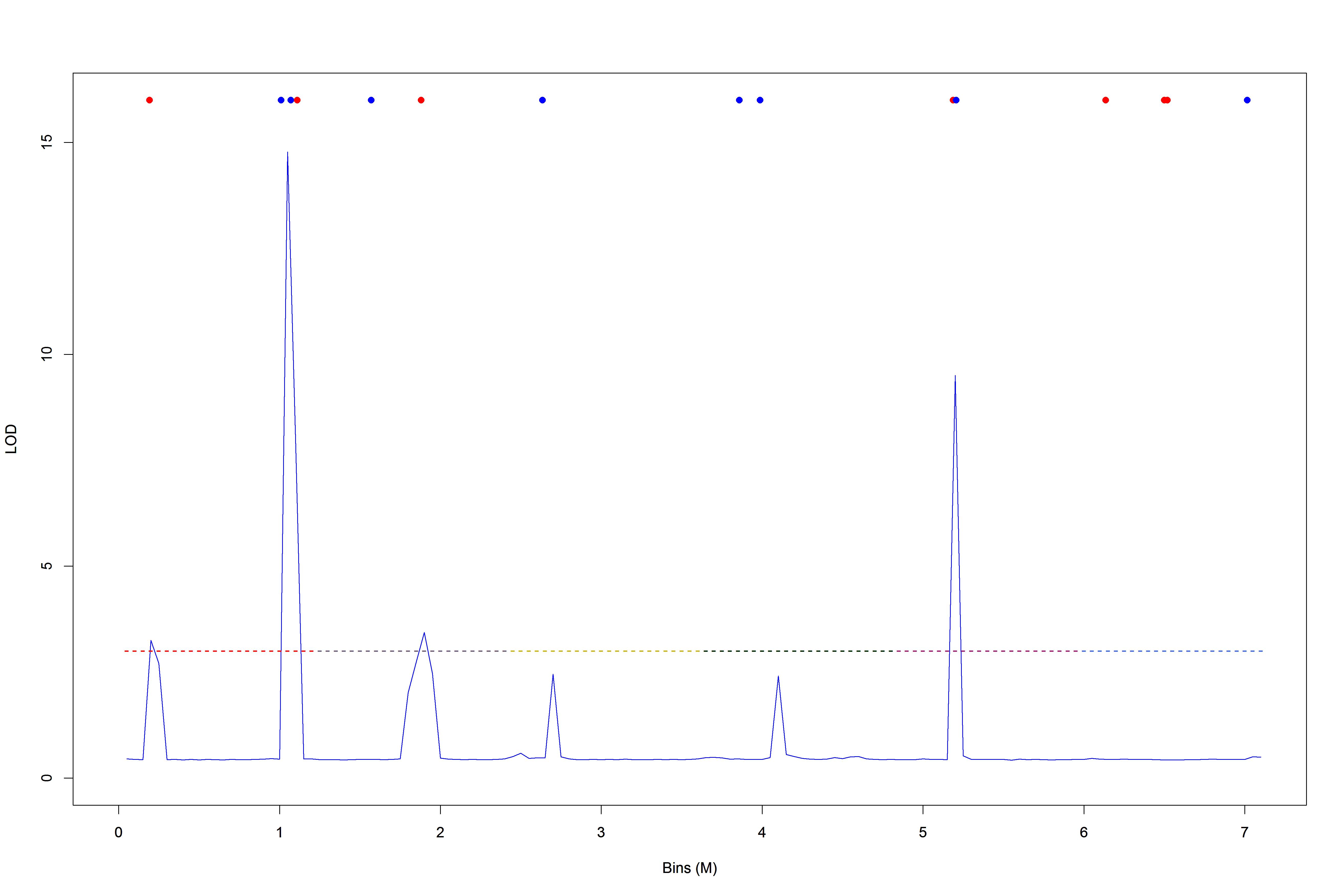

Supplement: Additional file 1: Figure S1. — Genomic profile for QTL in seven simulated linkage groups using RJMCMC for 400 markers. The red dots represent the non-epistatic QTL, and the blue dots represent the simulated epistatic QTL. The dotted line represents the LOD criterion (LOD=3). (JPEG 425 kb) [file 12864_2016_3342_MOESM1_ESM.jpeg]

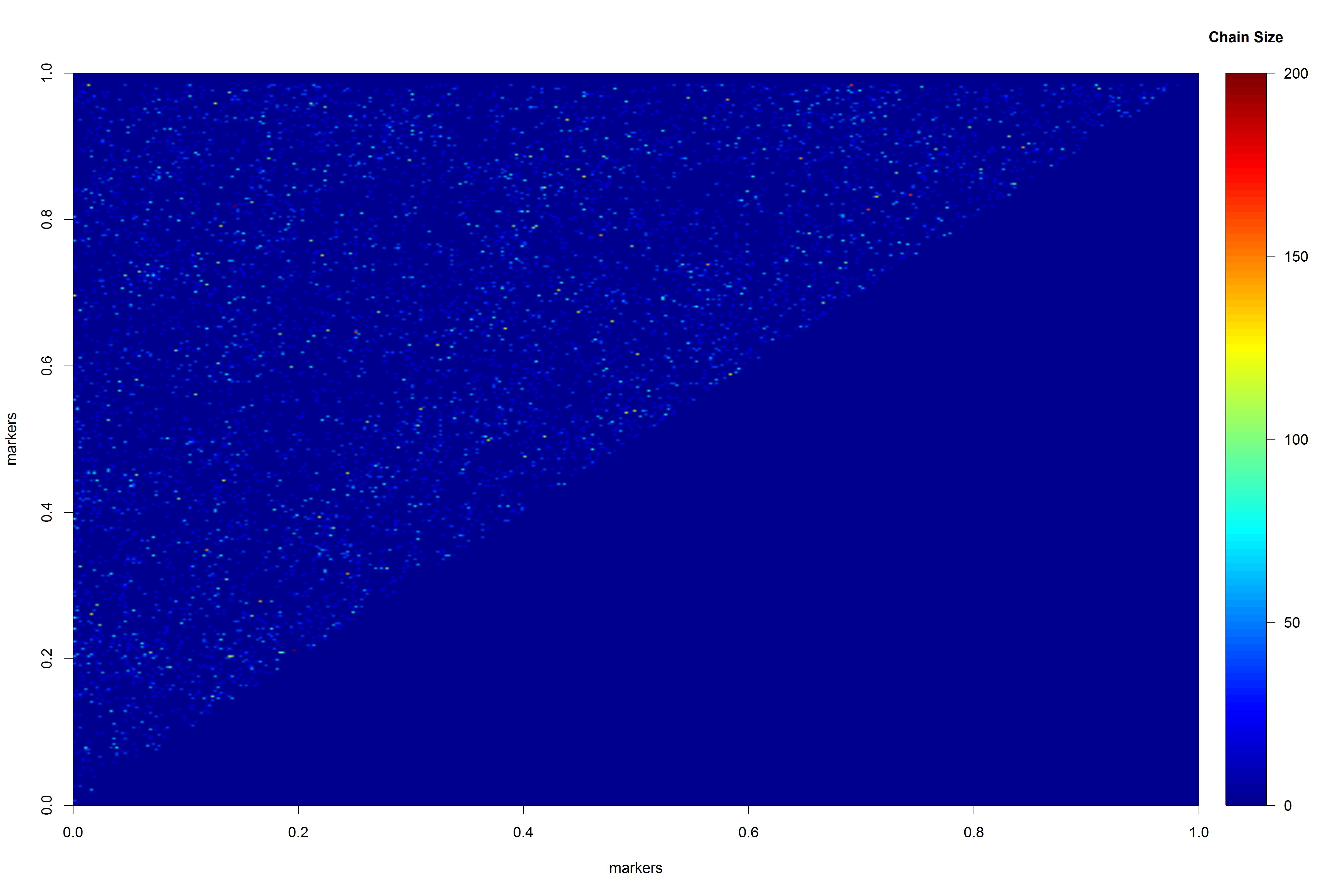

Supplement: Additional file 2: Figure S2. — Heat map for 79,800 epistatic QTL and chain size in the reversible jumping process. The more red that the epistatic intensity is, the more time the QTL was retained in the MCMC process, meaning its effect on the epistatic complex is more likely. (JPEG 570 kb) [file 12864_2016_3342_MOESM2_ESM.jpeg]

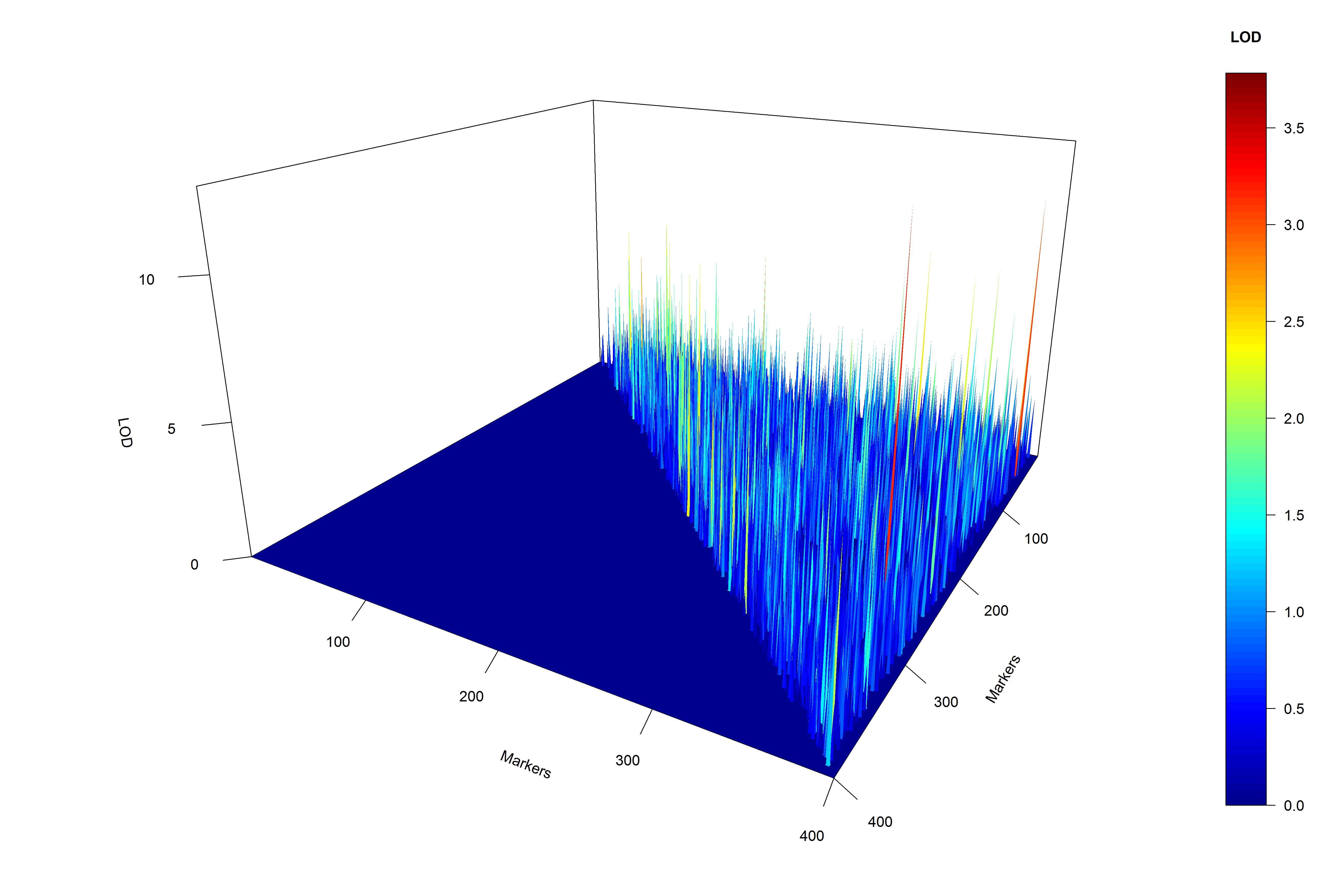

Supplement: Additional file 3: Figure S3. — LOD plot of 79,800 raw epistatic combinations. (JPEG 864 kb) [file 12864_2016_3342_MOESM3_ESM.jpeg]

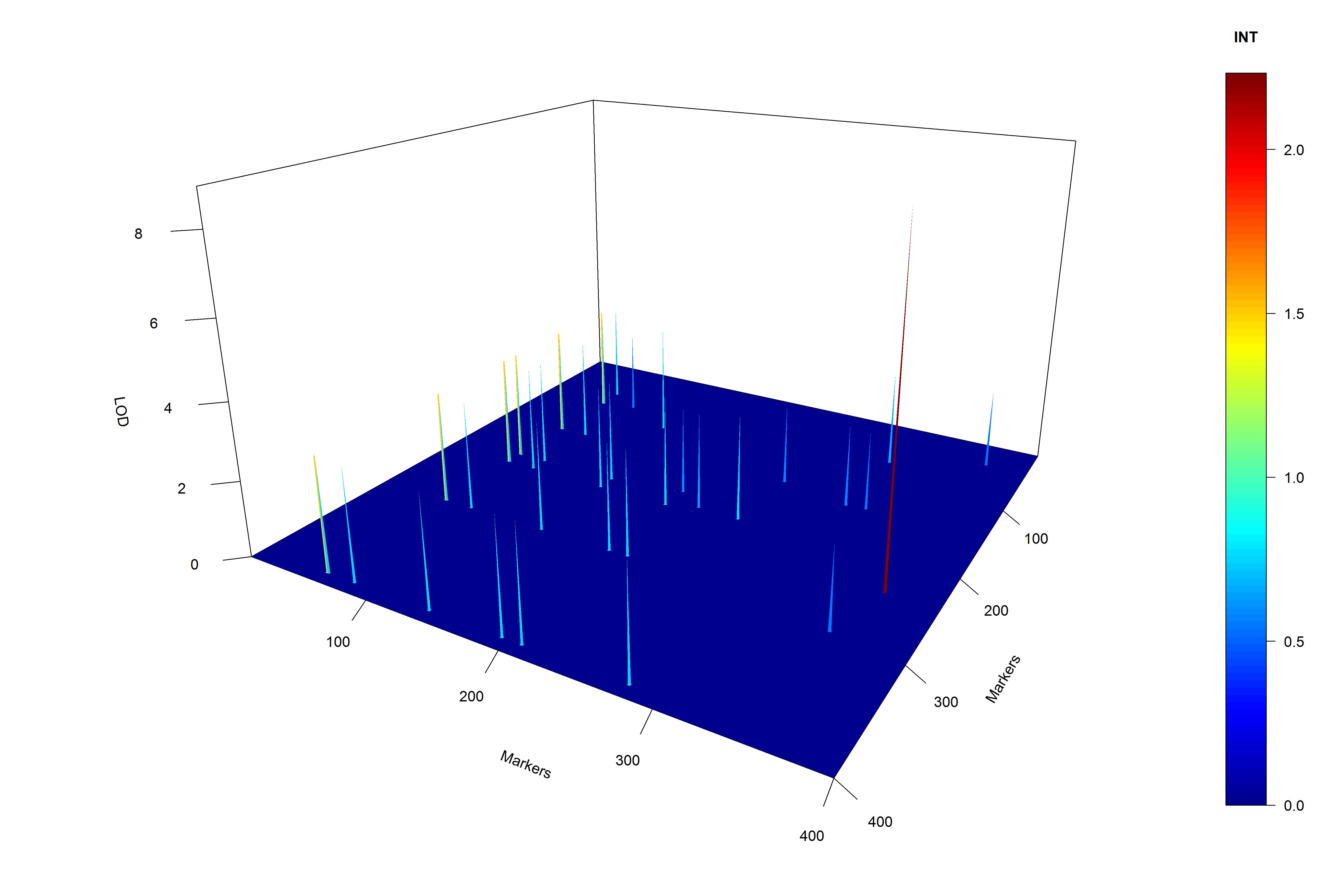

Supplement: Additional file 4: Figure S4. — 3D plot resolution for the 79,800 weighted epistatic combinations obtained by RJMCMC. The lower triangular plot represents the simulated epistatic effect, and the upper plot represents the estimated epistatic effect. (JPEG 483 kb) [file 12864_2016_3342_MOESM4_ESM.jpeg]
