# Supplementary material for: Bayesian reversible-jump for epistasis analysis in genomic studies
Source: BMC Genomics. 2016 Dec 9;17:1012. doi: 10.1186/s12864-016-3342-6 (PMC5148921; doi:10.1186/s12864-016-3342-6)
Supplement: Additional file 5: Table S1. — Selected epistases presenting with LOD scores greater than or equal to 3.00 and with chain sizes larger than 500 obtained in the simulated data from 400 markers. Sixteen QTL were simulated, and the first eight were combined pairwise, totaling 28 epistatic interactions. The epistatic QTL were named 58, 57, 80, 140, 208, 194, 279 and 389. (DOCX 13 kb) [file 12864_2016_3342_MOESM5_ESM.docx]

Selected epistasis presenting LOD score >=3.00 and chain size larger than 500 obtained in the simulated data from 400 markers. Sixteen QTL were simulated and the first eight were combined in pairwise totaling 28 epistatic interaction The epistatic QTLwere named as 58, 57, 80, 140, 208, 194, 279 and 389.

| **Selected Epistasis** | **Simulated*** | **Dist1** | **Dist2** |
| --- | --- | --- | --- |
| 200x216 | 194x208 | 0.03 | 0.14 |
| 291x352 | 279x389 | 0.11 | 0.634 |
| 144x317 | - | - | - |
| 35x370 | 57x389 | 0.3 | 0.25 |
| 75x84 | - | - | - |
| 135x251 | 140x279 | 0.1 | 0.34 |
| 146x306 | 140x279 | 0.11 | 0.37 |
| 98x127 | 80x140 | 0.22 | 0.21 |
| 185x195 | - | - | - |
| 201x247 | - | - | - |
| 67x306 | - | - | - |
| 237x365 | - | - | - |

* Simulated QTL closest from the estimated. Dist1 is the Kosambi distance (cM) from the first marker and Dist2 is the distance from the second marker.
