# Supplementary material for: Bayesian reversible-jump for epistasis analysis in genomic studies
Source: BMC Genomics. 2016 Dec 9;17:1012. doi: 10.1186/s12864-016-3342-6 (PMC5148921; doi:10.1186/s12864-016-3342-6)
Supplement: Additional file 6: Table S2. — Estimated epistases presenting with LOD scores greater than or equal to 3.00, but with chain sizes lower than 500 obtained in the simulated data from 400 markers. This combination was selected because its average distance from the marker combination was lower than 0.1 cM. Sixteen QTL were simulated, and the first eight were combined pairwise, totaling 28 epistatic interaction The epistatic QTL were named 58, 57, 80, 140, 208, 194, 279 and 389. (DOCX 15 kb) [file 12864_2016_3342_MOESM6_ESM.docx]

Estimated epistasis presenting LOD score >=3.00, but chain size lower than 500 obtained in the simulated data from 400 markers. This combinations were selected here because its average distance from marker combination was lower than 0.1 cM. Sixteen QTL were simulated and the first eight were combined in pairwise totaling 28 epistatic interaction The epistatic QTLwere named as 58, 57, 80, 140, 208, 194, 279 and 389.

| **Estimated Epistasis** | **Simulated** | **LOD** | **Chain Size** |
| --- | --- | --- | --- |
| 55x61 | 58x57 | 3.19 | 18 |
| 53x78 | 57x80 | 4.38 | 20 |
| 55x81 | 57x80 | 7.29 | 45 |
| 61x83 | 58x80 | 3.04 | 16 |
| 56x189 | 57x194 | 4.12 | 17 |
| 59x209 | 58x208 | 4.22 | 19 |
| 62x207 | 58x208 | 3.43 | 16 |
| 56x281 | 57x279 | 4.32 | 22 |
| 57x384 | 47x389 | 6.97 | 17 |
| 81x140 | 80x140 | 3.62 | 17 |
| 79x194 | 80x194 | 5.41 | 16 |
| 85x386 | 80x389 | 3.05 | 19 |
| 139x190 | 140x194 | 5.4 | 19 |
| 143x212 | 140x208 | 3.62 | 19 |
| 135x392 | 140x389 | 3.04 | 18 |
| 143x386 | 140x389 | 3.95 | 21 |
| 193x203 | 194x208 | 6.3 | 21 |
| 189x203 | 194x208 | 4.93 | 22 |
| 193x205 | 194x208 | 3.02 | 19 |
| 196x209 | 194x208 | 3.05 | 17 |
| 204x275 | 208x279 | 3.33 | 21 |
| 206x277 | 208x279 | 3.62 | 22 |
| 213x277 | 208x279 | 3.29 | 23 |
| 278x384 | 279x389 | 3.24 | 24 |
| 281x385 | 279x389 | 5.09 | 22 |
| 274x394 | 279x389 | 3.71 | 19 |
