# Supplementary material for: Bayesian reversible-jump for epistasis analysis in genomic studies
Source: BMC Genomics. 2016 Dec 9;17:1012. doi: 10.1186/s12864-016-3342-6 (PMC5148921; doi:10.1186/s12864-016-3342-6)
Supplement: Additional file 7: Table S3 — Simulated QTL effects from the Qgene program. (DOCX 14 kb) [file 12864_2016_3342_MOESM7_ESM.docx]

Principal and epistatic QTL simulated from Qgene program.

| **QTL** | **Chomossome** | **Position** | **Chomossome** | **Position** | **a** | **d** | **aa** | **ad** | **da** | **dd** |
| --- | --- | --- | --- | --- | --- | --- | --- | --- | --- | --- |
| 1 | 1 | 42.2 |  |  | 2 | 2 | - | - | - | - |
| 2 | 2 | 4.3 |  |  | 2 | 2 | - | - | - | - |
| 3 | 2 | 23.0 |  |  | 2 | 2 | - | - | - | - |
| 4 | 3 | 93.7 |  |  | 2 | 2 | - | - | - | - |
| 5 | 5 | 61.3 |  |  | 2 | 2 | - | - | - | - |
| 6 | 5 | 111.0 |  |  | 2 | 2 | - | - | - | - |
| 19x25 | 2 | 16.7 | 2 | 82.0 | 0 | 0 | 0.15 | 0.2 | -0.1 | -1.7 |
| 54x72 | 4 | 32.2 | 5 | 90.6 | 0 | 0 | 1.9 | 0.54 | 1.32 | 0.23 |
| 59x91 | 4 | 92.8 | 6 | 102.6 | 0 | 0 | -0.45 | -1.12 | -0.14 | 0.98 |
| 59x94 | 4 | 92.8 | 6 | 106.5 | 0 | 0 | 1.54 | 0.25 | -1.84 | 0.54 |
